# Morbidity and measures of the diagnostic process in primary care for patients subsequently diagnosed with cancer

**DOI:** 10.1093/fampra/cmab139

**Published:** 2021-11-30

**Authors:** Minjoung M Koo, Ruth Swann, Sean McPhail, Gary A Abel, Cristina Renzi, Greg P Rubin, Georgios Lyratzopoulos

**Affiliations:** Epidemiology of Cancer Healthcare Outcomes (ECHO) Research Group, Research Department of Behavioural Science and Health, University College London, London, United Kingdom; National Disease Registration Service, NHS Digital, Leeds, West Yorkshire, United Kingdom; Epidemiology of Cancer Healthcare Outcomes (ECHO) Research Group, Research Department of Behavioural Science and Health, University College London, London, United Kingdom; National Disease Registration Service, NHS Digital, Leeds, West Yorkshire, United Kingdom; Cancer Research UK, London, United Kingdom; National Disease Registration Service, NHS Digital, Leeds, West Yorkshire, United Kingdom; Institute of Health Research, University of Exeter Medical School, St Luke’s Campus, Exeter, United Kingdom; Epidemiology of Cancer Healthcare Outcomes (ECHO) Research Group, Research Department of Behavioural Science and Health, University College London, London, United Kingdom; Population Health Sciences Institute, Newcastle University, Newcastle Upon Tyne, United Kingdom; Epidemiology of Cancer Healthcare Outcomes (ECHO) Research Group, Research Department of Behavioural Science and Health, University College London, London, United Kingdom; National Disease Registration Service, NHS Digital, Leeds, West Yorkshire, United Kingdom

**Keywords:** cancer, chronic disease, diagnosis, multimorbidity, primary care, risk assessment

## Abstract

**Background:**

There is uncertainty regarding how pre-existing conditions (morbidities) may influence the primary care investigation and management of individuals subsequently diagnosed with cancer.

**Methods:**

We identified morbidities using information from both primary and secondary care records among 11,716 patients included in the English National Cancer Diagnosis Audit (NCDA) 2014. We examined variation in 5 measures of the diagnostic process (the primary care interval, diagnostic interval, number of pre-referral consultations, use of primary care-led investigations, and referral type) by both primary care- and hospital records-derived measures of morbidity.

**Results:**

Morbidity prevalence recorded before cancer diagnosis was almost threefold greater using the primary care (75%) vs secondary care-derived measure (28%). After adjustment, there was limited variation in the primary care interval and the number of pre-referral consultations by either definition of morbidity. Patients with more severe morbidities were less likely to have had a primary care-led investigation before cancer diagnosis compared with those without any morbidity (adjusted odds ratio, OR [95% confidence interval]: 0.72 [0.60–0.86] for Charlson score 3+ vs 0; joint *P* < 0.001). Patients with multiple primary care-recorded conditions or a Charlson score of 3+ were more likely to have diagnostic intervals exceeding 60 days (aOR: 1.26 [1.10–1.45] and 1.19 [>1.00–1.41], respectively), and more likely to receive an emergency referral (aOR: 1.60 [1.26–2.02] and 1.61 [1.26–2.06], respectively).

**Conclusion:**

Among cancer cases with up to 2 morbidities, there was no evidence of differences in diagnostic processes and intervals in primary care but higher morbidity burden was associated with longer time to diagnosis and higher likelihood of emergency referral.

**Key Messages**

- Morbidity prevalence was 28% vs 75% based on hospital vs primary care records.
- Patients with up to 2 chronic conditions had comparable outcomes to those with none.
- For example, time from primary care to specialist referral did not vary by morbidity burden.
- However, patients with morbidities were more likely to be referred as an emergency.
- Patients with multiple or severe morbidities experienced longer time to diagnosis.

## Introduction

Pre-existing chronic conditions (morbidities) are common among the general population and among those diagnosed with cancer.^1–4^ Current evidence indicates individuals with morbidities are more likely to be diagnosed as an emergency (which is typically associated with poorer clinical outcomes and patient experience)^5^; more likely to be diagnosed with advanced cancer, although effects vary by specific morbidity^6^; and often experience longer intervals to treatment.^7^

The presence of pre-existing chronic conditions may influence the diagnostic process of cancer in primary care through complex mechanisms, resulting in a longer/shorter interval to diagnosis.^6^ Managing existing condition(s) could be prioritized over the appraisal of new symptoms caused by as yet undiagnosed cancer (the “competing demands” hypothesis); or bias the interpretation of new symptoms, particularly if they could plausibly be related to the morbidity (the “alternative explanations” hypothesis), both contributing to diagnostic overshadowing.^8,9^ Conversely, individuals with pre-existing morbidities may be more likely to have an incidental and quicker diagnosis of cancer through routine monitoring (the “surveillance effect” hypothesis).^6^

Morbidities have been associated with lower likelihood of referral or longer time from presentation to referral (i.e. a longer primary care interval),^10,11^ lower likelihood of endoscopy,^12^ and a longer diagnostic interval.^8,13–16^ However, this evidence chiefly relates to colorectal and lung cancer populations, with limited research on other cancers. Furthermore, information on pre-existing conditions is typically extracted from either primary care records alone, which could underestimate specialist managed conditions, or hospital records alone, which could underestimate less severe conditions.

Therefore, we aimed to describe morbidity prevalence among cancer patients who presented in primary care before diagnosis using 2 definitions of morbidity (as derived from either primary or secondary care records), and examine variation in measures of the diagnostic process in primary care by morbidity type.

## Methods

### Data and study population

We examined data from the National Cancer Diagnosis Audit (NCDA), described previously.^17^ Participating General Practitioners (GPs) and other primary care professionals extracted information from patient records on the diagnostic process for individuals diagnosed with cancer in 2014, as identified by cancer registrations from Public Health England’s National Cancer Registration and Analysis Service (NCRAS).

We excluded subsequent records of individuals who had multiple tumours, those diagnosed via screening (as auditors were not required to submit information for these patients), patients who did not present in primary care prior to diagnosis, patients aged <35 years old, and those with missing information on morbidity status (see Supplementary Material S1 for sample derivation).

### Measuring morbidity and other covariates of interest

Primary care-derived information on morbidities was available from the NCDA, which collected yes/no responses to whether each patient had any of the following^11^ conditions prior to cancer diagnosis: hypertension, cardiovascular disease, arthritis/musculo-skeletal disease, diabetes, chronic obstructive pulmonary disease or other chronic respiratory illness, cerebrovascular disease, cognitive impairment, longstanding physical disability, previous cancer, severe longstanding mental illness, or other [unspecified] comorbidity. Patients could therefore have no morbidities, a single morbidity, or 2 or more morbidities in any combination. The resulting number of NCDA conditions was used to describe primary care-derived morbidity burden (categorized as 0, 1, 2, 3+ NCDA morbidities).

Secondly, Charlson Comorbidity Index scores (hereafter referred to as the Charlson score) were produced using hospital inpatient data for the NCDA population by NCRAS.^18^ Diagnostic fields were used to identify Charlson score relevant conditions recorded in the 78 to 6 months (6 years) prior to diagnosis and derive a weighted score using previously published methodology.^19^ Each patient’s Charlson score was used to describe hospital-derived morbidity burden (indicated by a score of 0, 1, 2, or 3+).

Information was available from the NCDA on sex (male or female); age group (35–44, 45–54, 55–64, 65–74, 75–84, and 85+ years); ethnicity (white, non-white, and unknown); deprivation group (quintiles of Index of Multiple Deprivation [IMD] income domain scores, where 1 indicated least deprived and 5 indicated most deprived); and cancer type based on ICD-10 codes (categorized as 28 cancers).

### Outcomes of interest

We examined several measures of the diagnostic process in primary care for cancer, which we have described interchangeably as “diagnostic measures” in the text for brevity. Specifically, we focussed on the primary care interval and 2 correlated measures, the number of pre-referral consultations and the diagnostic interval. The primary care interval was defined as the number of days from first relevant presentation in primary care to the day of first specialist referral, while the diagnostic interval was defined as the time from first relevant presentation in primary care to diagnosis, in line with the Aarhus statement.^20^ The date of first relevant presentation was defined in the audit questionnaire as “the date when the patient first presented with symptoms ultimately attributed to the cancer diagnosis” while the date of diagnosis was established by NCRAS using European Network of Cancer Registries rules.^21^ Pre-referral diagnostic consultations were defined as the number of consultations from first relevant consultation until referral and parameterized as 1–2 consultations, or 3+ consultations.^22^

We also considered how morbidities may be associated with the use of investigations in primary care and type of referral. Information on use of primary care-led investigations prior to cancer diagnosis was collected through yes/no answers to a list of specific investigations in the NCDA, and parameterized as no investigations vs 1+ investigations.

Information on referral type was collected in the NCDA as one of the following options: 2-week-wait (2WW, fast-track specialist referrals for suspected cancer from primary care in England); urgent (expedited specialist referrals but not for suspected cancer); routine (non-urgent referrals); emergency referrals (same-day referrals to specialist services; these are typically associated with poorer cancer outcomes^5^); referrals to private hospitals; patient self-referrals to emergency services; other/unknown referral type; and screen-detected referral (after removing screen-detected cases based on final route to diagnosis, there remained 14 patients who were described as having been referred following screening). We aggregated responses into 3 categories: 2-week-wait; non-2WW (routine, urgent non-cancer, and private referrals); and emergency referrals. The remaining responses (*n* = 1,013, 9%) were excluded from consideration when examining referral type, namely: patient self-referrals to emergency services (*n* = 575); other or unknown referral type (*n* = 424); or screen-detected (*n* = 14). We considered a broader definition of emergency referrals including the 575 patients who had self-referred to emergency services in supplementary analyses (Supplementary Material S4).

### Statistical analysis

We described morbidity burden (number of NCDA morbidities: 0, 1, 2, 3+ and Charlson score: 0, 1, 2, 3+) among the study population. We then examined how morbidities were associated with each of the outcomes of interest (primary care interval, diagnostic interval, number of pre-referral consultations, investigations, and referral type) through descriptive statistics and logistic regression.

Specifically, for each outcome of interest we described centiles (for the primary care interval and diagnostic interval) or proportions (for pre-referral consultations, investigations and referral type), testing significance with Kruskal–Wallis tests comparing medians and Chi-squared tests, respectively. Subsequently, logistic regression was used to examine each association adjusting for covariates of interest. The 2 interval measures were dichotomized as ≤28 vs >28 days for the primary care interval, and ≤60 vs >60 days for the diagnostic interval. Two multivariate models were run for each outcome: the first adjusted for sex, age, ethnicity, and IMD; and the second additionally adjusting for cancer site. The events per variable criterion used for sample size considerations was satisfied for all models.^23^

### Supplementary analyses

We undertook several supplementary analyses described below:

- As we were interested in clinical decision making, in the main analysis we focus on patients referred as an emergency by the GP, but we additionally included patient self-referral to emergency services (Supplementary Material S4).
- Beyond considering morbidity count in the main analysis, we considered specific morbidity combinations for the 5 most common NCDA conditions (Supplementary Material S5).
- Additional to adjusting for cancer site in the main analysis, we re-ran the analysis restricted to patients with cancers stratified by diagnostic difficulty group. Three groups (stratified models) were used: low (cancers characterized by narrow symptom signatures with typically high positive predictive values [PPVs]), medium (broad symptom signature with mixed PPV values), and hard (broad symptom signature and low PPV values)^24^ (Supplementary Material S6).
- As existing evidence is concentrated in colorectal and lung cancer populations, we examined associations between morbidity and diagnostic measures in these 2 specific populations (Supplementary Material S7).
- Lastly, we examined associations between morbidity and a further (sixth) diagnostic measure, the referral-to-diagnosis interval (RDI; calculated by subtracting the primary care interval from the diagnostic interval) (Supplementary Material S8).

All analyses were conducted using Stata SE version 15.1 (StataCorp, College Station, TX, 2017).

## Results

### Study population

Of the 11,716 cancer patients in our study population, 53% were men, the majority were white (88%), and aged 65+ years (66%) (Supplementary Material S2). The most common cancer diagnoses in the study population were prostate (15%), breast (13%), and lung (12%) cancer. Hypertension was the most common morbidity while physical disability was the least common (reported among 39% and 1% of patients, respectively).

Three quarters of the study population (8,844/11,716, 75%) had 1 or more morbidities noted in primary care. In comparison, based on hospital-derived Charlson scores, 28% of the study population had 1 or more morbidities. Cross-tabulation of the NCDA morbidity count excluding non-Charlson conditions (hypertension, physical disability, and severe mental illness) and Charlson score indicated differences in overlap between the 2 measures (Supplementary Material S3). Specifically, 94% of patients with no morbidities noted in the NCDA also had a Charlson score of zero indicating no conditions recorded in hospital records. Conversely however, over half (54%) of patients who had a Charlson score of zero (no conditions) had at least 1 morbidity recorded in primary care.

### Morbidities and diagnostic measures

#### Primary care and diagnostic interval

Among the study population, the median (interquartile range [IQR]) primary care interval was 5 (0–27) days, while the median (IQR) diagnostic interval was 42 (18–91) days. For both definitions of morbidity, cancer patients with 1 or more conditions tended to have slightly longer primary care and diagnostic intervals than those without (Table 1). However, there was no evidence to support variation in the primary care interval by either morbidity definition when adjusting for age, sex, ethnicity, deprivation, and cancer type.

**Table 1.** Descriptive statistics and logistic regression examining distribution of the primary care interval (PCI) and diagnostic interval (DI) and likelihood of a PCI >28 days and DI >60 days, by NCDA morbidity count and Charlson scores among 11,716 patients diagnosed with cancer in England in 2014.

	*N* ^a^	Centiles of the PCI/DI (days)					PCI >28 days	Crude OR for PCI >28 days or DI >60 days	Adjusted OR^d^ for PCI >28 days or DI >60 days	Adjusted OR^e^ for PCI >28 days or DI >60 days
		10th	25th	50th	75th	90th	*N* (%)	OR (95% CI)	OR (95% CI)	OR (95% CI)
Primary care interval										
All patients	9,402	0	0	5	27	77	2,246 (24%)	—	—	—
No. of NCDA morbidities		0.001^b^						0.073^c^	0.102^c^	0.369^c^
0 morbidities	2,342	0	0	3	23	77	518 (22%)	Ref	Ref	Ref
1 morbidity	2,922	0	0	5	28	82	716 (25%)	1.14 (>1.00–1.30)	1.13 (0.99–1.29)	1.08 (0.94–1.24)
2 morbidities	2,274	0	0	5	27	66	539 (24%)	1.09 (0.95–1.25)	1.09 (0.94–1.27)	1.06 (0.91–1.24)
3+ morbidities	1,864	0	0	5	29	84	473 (25%)	1.20 (1.04–1.38)	1.21 (1.04–1.42)	1.16 (0.98–1.36)
Charlson score		0.947^b^						0.627^c^	0.590^c^	0.490^c^
0	6,872	0	0	5	27	78	1,646 (24%)	Ref	Ref	Ref
1	1,265	0	0	5	25	73	288 (23%)	0.94 (0.81–1.08)	0.92 (0.80–1.07)	0.90 (0.78–1.05)
2	669	0	0	4	29	71	170 (25%)	1.08 (0.90–1.30)	1.07 (0.89–1.29)	1.06 (0.87–1.28)
3+	596	0	0	6	27.5	67	142 (24%)	0.99 (0.82–1.21)	0.97 (0.80–1.19)	0.96 (0.78–1.18)
Diagnostic interval										
All patients	10,236	10	18	42	91	190	3,831 (37%)	—	—	—
No. of NCDA morbidities		<0.001^b^						<0.001^c^	0.001^c^	0.004^c^
0 morbidities	2,476	9	15	38	82	177	861 (35%)	Ref	Ref	Ref
1 morbidity	3,171	10	19	44	93	195	1,216 (38%)	1.17 (1.05–1.30)	1.15 (1.02–1.29)	1.11 (0.98–1.25)
2 morbidities	2,485	10	18	42	85	182	902 (36%)	1.07 (0.95–1.20)	1.06 (0.93–1.20)	1.04 (0.91–1.19)
3+ morbidities	2,104	10	20	47	100	209	852 (40%)	1.28 (1.13–1.44)	1.29 (1.13–1.48)	1.26 (1.10–1.45)
Charlson score		0.016^b^						0.044^c^	0.092^c^	0.055^c^
0	7,404	10	18	42	87	181	2,721 (37%)	Ref	Ref	Ref
1	1,408	9	18	42.5	95.5	209	530 (38%)	1.04 (0.92–1.17)	1.03 (0.91–1.16)	1.02 (0.90–1.15)
2	747	10	20	47	107	212	305 (41%)	1.19 (1.02–1.38)	1.17 (>1.00–1.37)	1.19 (1.01–1.40)
3+	677	10	19	46	106	213	275 (41%)	1.18 (>1.00–1.38)	1.16 (0.99–1.37)	1.19 (>1.00–1.41)

^a^Total *n* = 9,402, 80% of patients had complete information on the PCI and *n* = 10,236, 87% of patients had complete information on the DI.

^b^Kruskal–Wallis test *P* value at 50th centile.

^c^Wald test for overall significance.

^d^Model including measure of morbidity, sex, age, ethnicity, and IMD.

^e^Model including measure of morbidity, sex, age, ethnicity, IMD, and cancer.

In contrast, patients who had 3+ primary care morbidities were more likely to experience long diagnostic intervals than those with no morbidities (odds ratio, OR [95% confidence interval, CI]: 1.26 [1.10–1.45]). Similarly, patients with more severe conditions (Charlson score of 2 or 3+) had longer intervals on average than those with no morbidities (OR [95% CI]: 1.19 [1.01–1.40] and 1.19 [>1.00–1.41], respectively, Table 1). Findings from quantile regression (a parametric approach suitable for skewed continuous outcome data) for the primary care interval and diagnostic interval indicated comparable findings (data not shown).

#### Number of pre-referral consultations

A slightly higher proportion of patients with 1 or more primary care or hospital-derived morbidities had 3+ consultations prior to referral compared with individuals without morbidities (Table 2). Multivariate analysis indicated patients with high morbidity count were more likely to have 3+ consultations compared with those with no morbidities (OR [95% CI]: 1.21 [1.05–1.40], joint *P* value = 0.010) but there was no evidence for variation in odds of 3+ consultations by hospital-derived morbidity.

**Table 2.** Descriptive statistics and logistic regression examining proportion and likelihood of having 3 or more pre-referral consultations in primary care by NCDA morbidity count and Charlson scores among 11,716 patients diagnosed with cancer in England in 2014.

	*N* ^a^	Three or more consultations *N* (%)	Crude OR for 3 or more consultations OR (95% CI)	Adjusted OR^c^ for 3 or more consultations OR (95% CI)	Adjusted OR^d^ for 3 or more consultations OR (95% CI)
All patients	11,473	2,837 (25%)	—	—	—
No. of NCDA morbidities			<0.001^b^	<0.001^b^	0.010^b^
0 morbidities	2,839	599 (21%)	Ref	Ref	Ref
1 morbidity	3,554	880 (25%)	1.23 (1.09–1.38)	1.11 (0.98–1.25)	1.03 (0.91–1.17)
2 morbidities	2,760	675 (24%)	1.21 (1.07–1.37)	1.04 (0.91–1.19)	0.98 (0.85–1.13)
3+ morbidities	2,320	683 (29%)	1.56 (1.37–1.77)	1.31 (1.14–1.51)	1.21 (1.05–1.40)
Charlson score			0.05^b^	0.896^b^	0.982^b^
0	8,298	1,997 (24%)	Ref	Ref	Ref
1	1,561	405 (26%)	1.11 (0.98–1.25)	1.03 (0.91–1.17)	0.98 (0.86–1.12)
2	844	224 (27%)	1.14 (0.97–1.34)	1.04 (0.89–1.23)	0.98 (0.83–1.16)
3+	770	211 (27%)	1.19 (1.01–1.41)	1.05 (0.88–1.24)	0.97 (0.82–1.16)

^a^Total *n* = 11,473, 98% of patients had complete information on number of pre-referral consultations.

^b^Wald test for overall significance.

^c^Model including measure of morbidity, sex, age, ethnicity, and IMD.

^d^Model including measure of morbidity, sex, age, ethnicity, IMD, and cancer.

#### Primary care-led investigations

Three-fifths (60%) of patients had 1+ investigations in primary care prior to cancer diagnosis with this proportion being higher among individuals with primary care-derived morbidities, while there was little difference by hospital-derived morbidities (Table 3). Multivariate analysis indicated no association between primary care-derived morbidity count and odds of investigation, whereas those with hospital-derived morbidities were less likely to be investigated (OR [95% CI]: 0.74 [0.65–0.85] and 0.72 [0.60–0.86] for patients with a Charlson score of 1 and 3+ vs 0, respectively).

**Table 3.** Descriptive statistics and logistic regression examining proportion and likelihood of having 1 or more primary care-led investigations by NCDA morbidity count and Charlson scores among 11,716 patients diagnosed with cancer in England in 2014.

	*N* ^a^	At least 1 investigation *N* (%)	Crude OR for at least 1 investigation OR (95% CI)	Adjusted OR^c^ for at least 1 investigation OR (95% CI)	Adjusted OR^d^ for at least 1 Investigation OR (95% CI)
Total	11,230	6,777 (60%)	—	—	—
No. of NCDA morbidities			<0.001^b^	0.914^b^	0.365^b^
0 morbidities	2,762	1,544 (56%)	Ref	Ref	Ref
1 morbidity	3,487	2,128 (61%)	1.24 (1.12–1.37)	1.01 (0.90–1.12)	0.91 (0.80–1.04)
2 morbidities	2,703	1,678 (62%)	1.29 (1.16–1.44)	0.97 (0.86–1.10)	0.89 (0.77–1.03)
3+ morbidities	2,278	1,427 (63%)	1.32 (1.18–1.48)	0.97 (0.85–1.11)	0.88 (0.76–1.03)
Charlson score			0.630^b^	0.004^b^	<0.001^b^
0	8,102	4,886 (60%)	Ref	Ref	Ref
1	1,541	917 (60%)	0.97 (0.87–1.08)	0.84 (0.75–0.95)	0.74 (0.65–0.85)
2	832	518 (62%)	1.09 (0.94–1.26)	0.93 (0.80–1.08)	0.87 (0.73–1.04)
3+	755	456 (60%)	>1.00 (0.86–1.17)	0.80 (0.68–0.94)	0.72 (0.60–0.86)

^a^Total *n* = 11,230, 96% of patients had complete information on investigations.

^b^Wald test for overall significance.

^c^Model including measure of morbidity, sex, age, ethnicity, and IMD.

^d^Model including measure of morbidity, sex, age, ethnicity, IMD, and cancer.

#### Referral type

The proportion of patients referred as an emergency increased with an increasing number of primary care or hospital-derived morbidities (Table 4). Multivariate findings indicated higher odds of emergency referral among those with a greater burden of morbidity: OR (95% CI): 1.60 (1.26–2.02) for patients with 3+ NCDA morbidities and 1.61 (1.26–2.06) for patients with a Charlson score of 3+ compared with those with no morbidities.

**Table 4.** Descriptive statistics and logistic regression examining referral type and likelihood of emergency referral by NCDA morbidity count and Charlson scores among 11,716 patients diagnosed with cancer in England in 2014.

	Total *N*^a^	Referral type					
		2WW	Non-2WW	Emergency	Crude OR for emergency referral	Adj OR^c^ for emergency referral	Adj OR^d^ for emergency referral
		*N* (%)	*N* (%)	*N* (%)	OR (95% CI)	OR (95% CI)	OR (95% CI)
Total	10,703	7,785 (73%)	1,928 (18%)	990 (9%)	—	—	—
No. of NCDA morbidities					<0.001^b^	<0.001^b^	<0.001^b^
0 morbidities	2,686	2,001 (74%)	518 (19%)	167 (6%)	Ref	Ref	Ref
1 morbidity	3,346	2,436 (73%)	626 (19%)	284 (8%)	1.40 (1.15–1.71)	1.24 (1.01–1.53)	1.14 (0.92–1.42)
2 morbidities	2,550	1,873 (73%)	419 (16%)	258 (10%)	1.70 (1.39–2.08)	1.36 (1.09–1.69)	1.28 (1.02–1.61)
3+ morbidities	2,121	1,475 (70%)	365 (17%)	281 (13%)	2.30 (1.88–2.82)	1.72 (1.37–2.15)	1.60 (1.26–2.02)
Charlson score					<0.001^b^	<0.001^b^	<0.001^b^
0	7,786	5,796 (74%)	1,378 (18%)	612 (8%)	Ref	Ref	Ref
1	1,469	1,005 (68%)	282 (19%)	182 (12%)	1.66 (1.39–1.98)	1.48 (1.24–1.77)	1.41 (1.16–1.70)
2	773	535 (69%)	150 (19%)	88 (11%)	1.51 (1.19–1.91)	1.30 (1.02–1.66)	1.21 (0.94–1.56)
3+	675	449 (67%)	118 (17%)	108 (16%)	2.23 (1.79–2.79)	1.80 (1.43–2.26)	1.61 (1.26–2.06)

^a^Total *n* = 10,703. 2WW = 2-week-wait urgent referrals for suspected cancer; non-2WW includes routine, urgent non-cancer, and private referrals; emergency includes emergency referrals; 1,013 (9%) individuals whose referrals were categorized as patient self-referrals to A&E, “other,” “screen-detected,” and “not known” were excluded.

^b^Wald test for overall significance.

^c^Model including measure of morbidity, sex, age, ethnicity, and IMD.

^d^Model including measure of morbidity, sex, age, ethnicity, IMD, and cancer. No melanoma patients were referred as an emergency and so these individuals (*n* = 665) were excluded from the model.

**Fig. 1.**
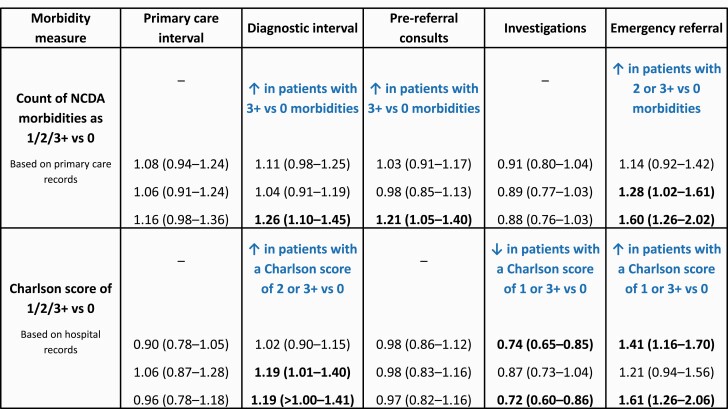
Summary of associations between morbidity (defined as NCDA conditions and Charlson scores) and measures of the diagnostic process in primary care among 11,716 cancer patients.

Adjusted associations between the 2 definitions of morbidity and the 5 examined diagnostic measures in primary care are summarized in Fig. 1.

### Supplementary analyses

When self-referrals to emergency services were additionally considered as emergency referrals, there was little difference in effect size or direction of associations compared with the main analysis (Supplementary Material S4).

Examining associations between specific primary care morbidity combinations, multimorbidity was more often associated with higher odds of a longer primary care and diagnostic interval, multiple pre-referral consultations, and emergency referrals compared with no morbidities in adjusted analyses not including cancer site. Adjusted models including cancer site indicated little evidence for variation in the examined diagnostic measures by specific morbidity status apart from emergency referral, where adjusted odds of emergency referral were consistently higher among patients with multimorbidity compared with those with no conditions (Supplementary Material S5).

When stratifying by cancer diagnostic difficulty, the number of morbidities was typically (but not always) associated with worse outcomes (e.g. higher odds of a long diagnostic interval, multiple pre-referral consultations, and emergency referral) among patients who were diagnosed with a cancer that had a broad symptom signature with mixed PPVs for cancer (“medium” diagnostic difficulty, e.g. colorectal cancer). This was less often observed among patients diagnosed with a cancer that had a narrow symptom signature (“easy” diagnostic difficulty, e.g. breast cancer) or a broad symptom signature of mostly low PPVs (“hard” diagnostic difficulty, e.g. brain cancer) (Supplementary Material S6).

Investigating associations between morbidity and diagnostic measures among colorectal cancer patients and lung cancer patients, we found no evidence to support variation in the examined diagnostic measures although colorectal cancer patients with multimorbidity were more likely to experience longer time to diagnosis (Supplementary Material S7).

Considering time to diagnosis after referral from primary care, the overall median (IQR) RDI was 25 (13–53) days, and the proportion of individuals with RDI longer than 28 days increased with morbidity count or Charlson score. Adjusted analyses indicated associations consistent with the diagnostic interval (Supplementary Material S8).

## Discussion

### Summary of findings

We examined associations between 2 definitions of morbidity burden and measures of the diagnostic process in primary care. Considering patients with up to 2 chronic conditions, associations with the primary care interval, pre-referral consultations, and investigations were modest and inconsistent. However, patients with a high burden of morbidity, measured either using primary care or secondary care records, had longer diagnostic intervals (which includes time under specialist management in addition to the primary care interval). Those with severe morbidity were less likely to receive pre-referral investigations in primary care, while both primary care and hospital-defined morbidity were associated with greater likelihood of emergency referral.

### Comparison to literature

Associations between morbidities and prolonged time to referral from primary care^10,11^ and lower likelihood of investigations^12^ for suspected cancer have previously been described in small populations (<600 patients). More recent and larger studies of colorectal and lung cancer patients have described associations between higher morbidity burden and prolonged time to diagnosis,^8,13–16^ and greater likelihood of emergency referral.^5,6,25^ Our findings of an association between morbidity with time to diagnosis and emergency referral are in line with those described in the literature (see Supplementary Material S7 for lung and colorectal cancer-specific analyses).

In contrast, in adjusted analyses we found limited variation by morbidity in the primary care interval, pre-referral consultations, and use of primary care-led investigations among those with 1 or 2 morbidities. Additionally, colorectal cancer patients with multiple primary care-derived morbidities were more likely to have a longer diagnostic interval though this was not seen for lung cancer patients, without evidence of variation in the other outcomes of interest by morbidity burden (Supplementary Material S8).

### Limitations and strengths

We studied a large and diverse cancer patient population identified through cancer registration. Capturing morbidity using 2 independent sources (primary care morbidity count and hospital record-based Charlson score) enabled a complementary description of morbidity burden.

Our findings arise from a unique source of clinically curated information relating to primary care events and processes that occur prior to cancer diagnosis. Some of the examined outcomes of interest depend on retrospectively judging whether a consultation was relevant to the subsequently diagnosed cancer. Relevance may be harder to judge in individuals who have a higher background rate of consultations (which could be more common among those with multimorbidity); however completed audit responses could not be validated due to the anonymous nature of data collection. Furthermore, we could not directly examine the influence of morbidity on cognitive tasks involved in diagnostic reasoning by clinicians which could enrich the interpretation of our findings; such a study would be resource-intensive given the relative rarity of cancer diagnosis in primary care.

Studying a larger study population could have enabled cancer- or symptom-specific analyses, which could account for differences in the presenting features of patients with different cancers.

### Interpretation and implications

Among patients with a low burden of morbidity, we found modest variation in several diagnostic measures (primary care interval, number of pre-referral consultations, and use of investigations), particularly among patients with 1 or 2 morbidities. This may reflect different explanations.

First, our findings may reflect heterogeneous effects of different morbidities, where certain morbidities increase, and others decrease, the likelihood of cancer suspicion (possibly variably for different cancer site).^6^ However, restricting the analysis to specific morbidities and their combinations did not support this hypothesis (Supplementary Material S5).

Second, the findings may indicate that a small number of morbidities exerts no measurable influence on the diagnostic measures. Given most patients have at least 1 chronic condition, primary care physicians are likely well accustomed to appraising new symptoms in patients with underlying chronic disease, which may explain the observed lack of associations.

Third, as the starting point of the primary care interval requires the first *relevant* consultation to be determined, any real differences in healthcare utilization between individuals with and without morbidity may hard to distinguish.^20,26^ For example, the starting point of both the primary care interval and diagnostic interval, namely the date of first relevant presentation, and the number of pre-referral consultations may be harder to identify among individuals with higher background consultation rates prior to cancer diagnosis (which may be due to their morbidities). Therefore, the limited variation could reflect methodological and conceptual challenges in assigning the timing of the first consultation with relevant symptoms in patients with chronic conditions.

Nevertheless, when we examined the diagnostic interval (which is defined with the same starting point as the primary care interval), we found that patients with a high burden of primary care or hospital-defined morbidity were more likely to experience longer time to diagnosis. This implies prolonged management of diagnostic processes within the hospital setting, for example due to people with multiple or severe morbidities requiring more complex preparation and risk assessment prior to invasive investigations such as endoscopies,^27^ and is supported by the observed variation in the referral-to-diagnosis (RDI) interval (Supplementary Material S8).

Furthermore, we found morbidities were consistently associated with greater likelihood of emergency referral from primary care. The greater likelihood of clinical complexity or acute deterioration among individuals with multiple or severe chronic conditions means that an emergency referral may be clinically appropriate.^28–30^ Appropriate cancer diagnostic pathways need to be developed for the large number of people with pre-existing morbidities, taking their clinical complexities into account.

## Conclusion

Our study of individuals diagnosed with cancer found that the diagnostic interval, but not the primary care interval, was longer among patients with greater morbidity burden. There was no evidence of variation in primary care-based diagnostic measures among those with up to 2 pre-existing conditions. Nevertheless, morbidity burden was associated with greater likelihood of emergency referral, while those with multiple or severe morbidity were more likely to have prolonged time to diagnosis. Given the findings, it is reasonable to suggest that both improvement efforts and future research in this field should target patients with multiple or severe morbidity, and explore the reasons for prolonged diagnostic intervals in specialist care.

## Supplementary Material

cmab139_suppl_Supplementary_Material
