# Supplementary material for: Morbidity and measures of the diagnostic process in primary care for patients subsequently diagnosed with cancer
Source: Fam Pract. 2021 Nov 30;39(4):623–32. doi: 10.1093/fampra/cmab139 (PMC9295610; doi:10.1093/fampra/cmab139)
Supplement: cmab139_suppl_Supplementary_Material [file cmab139_suppl_supplementary_material.docx]

Supplementary material

[S1 Sample derivation following application of exclusion criteria 2](#_Toc84441270)

[S2 Patient-level characteristics of the study population and number of NCDA-recorded morbidities 3](#_Toc84441271)

[S3 Morbidity burden defined by NCDA morbidity count and Charlson score 5](#_Toc84441272)

[Table S3.1 Prevalence of morbidity as defined by primary care derived number of conditions (NCDA morbidity count) or hospital derived Charlson Comorbidity Index score (Charlson score) 5](#_Toc84441273)

[Table S3.2 Comparison of the NCDA morbidity count and Charlson score 5](#_Toc84441274)

[S4 Emergency referrals including self-referral to A&E 6](#_Toc84441275)

[S5 Analysis by morbidity combination 7](#_Toc84441276)

[S5.1 Morbidities and primary care interval, by morbidity combination 8](#_Toc84441277)

[S5.2 Morbidities and diagnostic interval, by morbidity combination 9](#_Toc84441278)

[S5.3 Morbidities and pre-referral consultations, by morbidity combination 10](#_Toc84441279)

[S5.4 Morbidities and investigations, by morbidity combination 11](#_Toc84441280)

[S5.5 Morbidities and emergency referral, by morbidity combination 13](#_Toc84441281)

[S6 Analyses by cancer diagnostic difficulty 15](#_Toc84441282)

[S6.1 Morbidities and primary care interval, by cancer diagnostic difficulty 16](#_Toc84441283)

[S6.2 Morbidities and diagnostic interval, by cancer diagnostic difficulty 17](#_Toc84441284)

[S6.3 Morbidities and pre-referral consultations, by cancer diagnostic difficulty 18](#_Toc84441285)

[S6.4 Morbidities and investigations, by cancer diagnostic difficulty 19](#_Toc84441286)

[S6.5 Morbidities and emergency referral, by cancer diagnostic difficulty 20](#_Toc84441287)

[S7 Lung and colorectal cancer-specific analyses 21](#_Toc84441288)

[S7.1 Morbidities and primary care interval, among patients diagnosed with lung and colorectal cancer only 22](#_Toc84441289)

[S7.2 Morbidities and diagnostic interval, among patients diagnosed with lung and colorectal cancer only 24](#_Toc84441290)

[S7.3 Morbidities and pre-referral consultations, among patients diagnosed with lung and colorectal cancer only 26](#_Toc84441291)

[S7.4 Morbidities and investigations, among patients diagnosed with lung and colorectal cancer only 28](#_Toc84441292)

[S7.5 Morbidities and emergency referral, among patients diagnosed with lung and colorectal cancer only 30](#_Toc84441293)

[S8 The referral-to-diagnosis interval 32](#_Toc84441294)

[S8.1 Descriptive statistics of the referral-to-diagnosis interval (RDI) by NCDA measured morbidity and Charlson score, and logistic regression output examining the outcome of a RDI >28 days 33](#_Toc84441295)

##
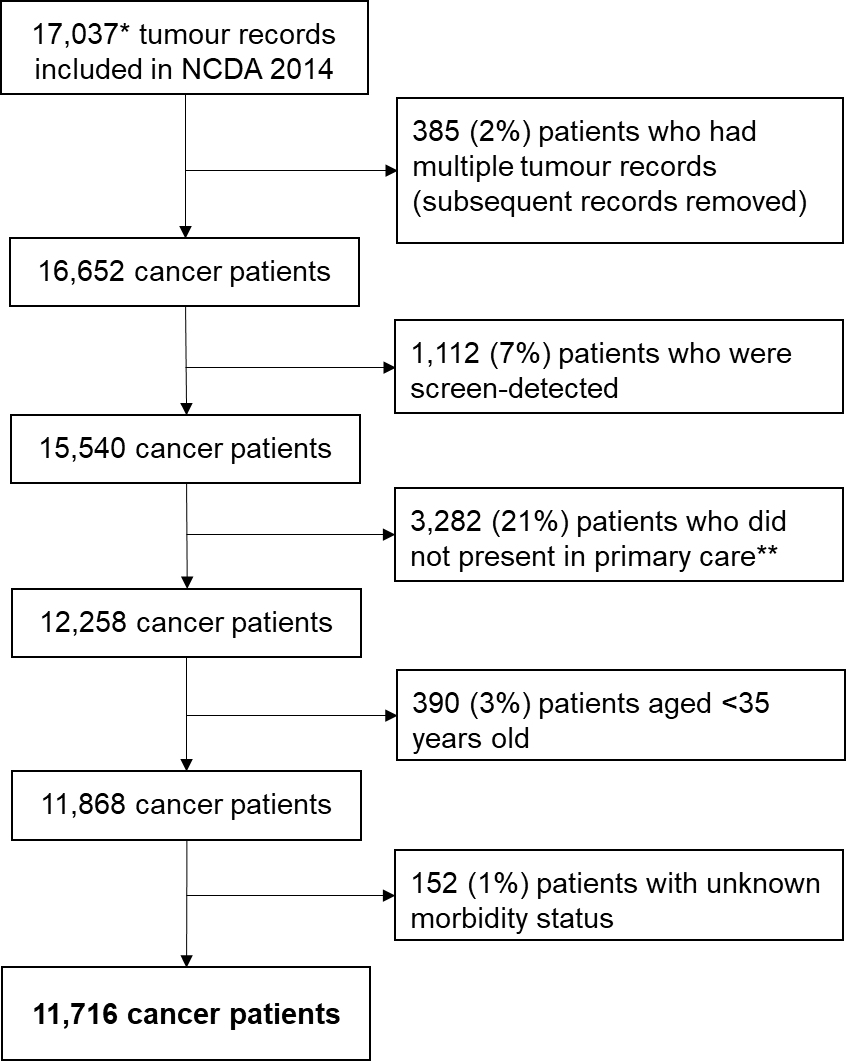
S1 Sample derivation following application of exclusion criteria

*This differs from the number of tumours described in the National Cancer Diagnosis Audit (NCDA) 2014 cohort profile published by Swann et al., 2018^[[1]](#footnote-1)^ (n=17042), due to five tumours no longer meeting the criteria of the NCDA subsequent to publication (due to being identified as duplicates, benign tumours, or not having been diagnosed in 2014).

**primary care defined as including GP practice, GP home visit, other primary care facility, telephone consultation, electronic consultation (email or video), community care nurse.

## S2 Patient-level characteristics of the study population and number of NCDA-recorded morbidities

|  |  | No. of NCDA morbidities | | | |
| --- | --- | --- | --- | --- | --- |
|  | N (% of total) | None | 1 | 2 | 3+ |
| Total | 11716 (100%) | 2872 (25%) | 3633 (31%) | 2819 (24%) | 2392 (20%) |
| Sex |  |  |  |  |  |
| Male | 6159 (53%) | 1348 (22%) | 1932 (31%) | 1554 (25%) | 1325 (22%) |
| Female | 5557 (47%) | 1524 (27%) | 1701 (31%) | 1265 (23%) | 1067 (19%) |
| Age group |  |  |  |  |  |
| 35-44 years | 497 (4%) | 349 (70%) | 119 (24%) | 25 (5%) | 4 (1%) |
| 45-54 years | 1203 (10%) | 671 (56%) | 379 (32%) | 107 (9%) | 46 (4%) |
| 55-64 years | 2126 (18%) | 755 (36%) | 753 (35%) | 418 (20%) | 200 (9%) |
| 65-74 years | 3446 (29%) | 696 (20%) | 1158 (34%) | 895 (26%) | 697 (20%) |
| 75-84 years | 3099 (26%) | 306 (10%) | 897 (29%) | 959 (31%) | 937 (30%) |
| 85+ years | 1345 (11%) | 95 (7%) | 327 (24%) | 415 (31%) | 508 (38%) |
| Ethnicity |  |  |  |  |  |
| White | 10302 (88%) | 2495 (24%) | 3185 (31%) | 2500 (24%) | 2122 (21%) |
| Non-white | 459 (4%) | 128 (28%) | 140 (31%) | 91 (20%) | 100 (22%) |
| Missing | 955 (8%) | 249 (26%) | 308 (32%) | 228 (24%) | 170 (18%) |
| IMD quintile |  |  |  |  |  |
| 1–least deprived | 2527 (22%) | 719 (28%) | 817 (32%) | 569 (23%) | 422 (17%) |
| 2 | 2636 (22%) | 693 (26%) | 865 (33%) | 596 (23%) | 482 (18%) |
| 3 | 2569 (22%) | 604 (24%) | 802 (31%) | 647 (25%) | 516 (20%) |
| 4 | 2123 (18%) | 483 (23%) | 644 (30%) | 517 (24%) | 479 (23%) |
| 5–most deprived | 1861 (16%) | 373 (20%) | 505 (27%) | 490 (26%) | 493 (26%) |
| Cancer |  |  |  |  |  |
| Prostate | 1775 (15%) | 431 (24%) | 587 (33%) | 447 (25%) | 310 (17%) |
| Breast | 1475 (13%) | 514 (35%) | 444 (30%) | 302 (20%) | 215 (15%) |
| Lung | 1460 (12%) | 212 (15%) | 417 (29%) | 409 (28%) | 422 (29%) |
| Colon | 870 (7%) | 174 (20%) | 265 (30%) | 221 (25%) | 210 (24%) |
| Melanoma | 678 (6%) | 243 (36%) | 189 (28%) | 139 (21%) | 107 (16%) |
| Lymphoma | 513 (4%) | 125 (24%) | 171 (33%) | 112 (22%) | 105 (20%) |
| Rectal | 498 (4%) | 140 (28%) | 141 (28%) | 118 (24%) | 99 (20%) |
| Other | 452 (4%) | 126 (28%) | 128 (28%) | 112 (25%) | 86 (19%) |
| Bladder | 374 (3%) | 54 (14%) | 122 (33%) | 103 (28%) | 95 (25%) |
| Oesophageal | 368 (3%) | 71 (19%) | 128 (35%) | 81 (22%) | 88 (24%) |
| Pancreatic | 360 (3%) | 68 (19%) | 113 (31%) | 104 (29%) | 75 (21%) |
| Endometrial | 346 (3%) | 90 (26%) | 92 (27%) | 98 (28%) | 66 (19%) |
| Renal | 322 (3%) | 63 (20%) | 100 (31%) | 78 (24%) | 81 (25%) |
| Leukaemia | 279 (2%) | 68 (24%) | 95 (34%) | 69 (25%) | 47 (17%) |
| CUP | 263 (2%) | 50 (19%) | 77 (29%) | 73 (28%) | 63 (24%) |
| Ovarian | 260 (2%) | 81 (31%) | 96 (37%) | 53 (20%) | 30 (12%) |
| Stomach | 231 (2%) | 50 (22%) | 72 (31%) | 51 (22%) | 58 (25%) |
| Oral/oropharyngeal | 200 (2%) | 61 (31%) | 73 (37%) | 32 (16%) | 34 (17%) |
| Myeloma | 185 (2%) | 32 (17%) | 74 (40%) | 38 (21%) | 41 (22%) |
| Liver | 164 (1%) | 19 (12%) | 43 (26%) | 51 (31%) | 51 (31%) |
| Mesothelioma | 118 (1%) | 22 (19%) | 42 (36%) | 33 (28%) | 21 (18%) |
| Brain/CNS | 107 (0.9%) | 38 (36%) | 33 (31%) | 16 (15%) | 20 (19%) |
| Laryngeal | 88 (0.8%) | 12 (14%) | 37 (42%) | 24 (27%) | 15 (17%) |
| Thyroid | 84 (0.7%) | 37 (44%) | 29 (35%) | 11 (13%) | 7 (8%) |
| Cervical | 58 (0.5%) | 27 (47%) | 16 (28%) | 10 (17%) | 5 (9%) |
| Small Intestine | 55 (0.5%) | 15 (27%) | 17 (31%) | 9 (16%) | 14 (25%) |
| Testicular | 54 (0.5%) | 35 (65%) | 12 (22%) | 3 (6%) | 4 (7%) |
| Vulval | 47 (0.4%) | 11 (23%) | 12 (26%) | 8 (17%) | 16 (34%) |
| Gallbladder | 32 (0.3%) | 3 (9%) | 8 (25%) | 14 (44%) | 7 (22%) |
| NCDA recorded morbidity | |  |  |  |  |
| Hypertension | 4542 (39%) | - | 1091 (24%) | 1580 (35%) | 1871 (41%) |
| CVD | 2354 (20%) | - | 370 (16%) | 708 (30%) | 1276 (54%) |
| Other morbidity | 2233 (19%) | - | 642 (29%) | 713 (32%) | 878 (39%) |
| MSK disease | 2113 (18%) | - | 428 (20%) | 681 (32%) | 1004 (48%) |
| Respiratory disease | 1697 (14%) | - | 379 (22%) | 514 (30%) | 804 (47%) |
| Diabetes | 1866 (16%) | - | 256 (14%) | 624 (33%) | 986 (53%) |
| Previous cancer | 1094 (9%) | - | 244 (22%) | 336 (31%) | 514 (47%) |
| Stroke | 769 (7%) | - | 59 (8%) | 213 (28%) | 497 (65%) |
| Cognitive impairment | 457 (4%) | - | 65 (14%) | 134 (29%) | 258 (56%) |
| Severe mental illness | 283 (2%) | - | 86 (30%) | 95 (34%) | 102 (36%) |
| Physical disability | 167 (1%) | - | 13 (8%) | 40 (24%) | 114 (68%) |

CBD: cerebrovascular disease; CNS: central nervous system; CUP: cancer of unknown primary; CVD: cardiovascular disease; IMD: index of multiple deprivation; MSK disease: arthritis/ musculo-skeletal disease; Respiratory disease: chronic respiratory illness

## S3 Morbidity burden defined by NCDA morbidity count and Charlson score

Morbidity prevalence measured as the number of morbidities (based on NCDA primary care data) and Charlson score (based on hospital data) among the study population are presented below. To improve comparability, three conditions were excluded from the NCDA count (hypertension, physical disability, and severe mental illness) as they are not included in the Charlson score.

### Table S3.1 Prevalence of morbidity as defined by primary care derived number of conditions (NCDA morbidity count) or hospital derived Charlson Comorbidity Index score (Charlson score)

|  | Number of chronic conditions (NCDA) / distribution of Charlson Comorbidity Index score (HES**)  Morbidity prevalence (no. of NCDA morbidities/ Charlson score) | | | |
| --- | --- | --- | --- | --- |
| Morbidity measure | 0 | 1 | 2 | 3+ |
| No. of chronic conditions (NCDA) | 2872 (25%) | 3633 (31%) | 2819 (24%) | 2392 (20%) |
| No. of chronic conditions (NCDA excluding non-Charlson conditions*) | 4091 (35%) | 4140 (35%) | 2347 (20%) | 1138 (10%) |
| Charlson score | 8431 (72%) | 1618 (14%) | 876 (7%) | 791 (7%) |

*excluding hypertension, physical disability, and severe mental illness which are not conditions included in the Charlson score

** based on information from HES inpatient hospital admission records within the last 6 years before the cancer diagnosis

### Table S3.2 Comparison of the NCDA morbidity count and Charlson score

In the first table, proportions are calculated by column. For example, among patients who were noted as having no morbidities in the NCDA, 94% also had a Charlson score of zero indicating no conditions. However among those with 3+ NCDA conditions, only 27% of patients had a Charlson score of 3+.

|  | No. of NCDA morbidities (excluding non-Charlson conditions*) | | | |
| --- | --- | --- | --- | --- |
| Charlson Comorbidity Index score | None | 1 condition | 2 conditions | 3+ conditions |
| Charlson score = 0 (no conditions) | 3841 (94%) | 3038 (73%) | 1206 (51%) | 346 (30%) |
| Charlson score = 1 | 157 (4%) | 639 (15%) | 529 (23%) | 293 (26%) |
| Charlson score = 2 | 68 (2%) | 284 (7%) | 337 (14%) | 187 (16%) |
| Charlson score = 3+ | 25 (1%) | 179 (4%) | 275 (12%) | 312 (27%) |

*excluding hypertension, physical disability, and severe mental illness which are not conditions included in the Charlson score

Proportions are calculated by row (i.e. denominator is those with a Charlson score). Therefore among patients with a Charlson score of zero, 46% of patients had no conditions recorded in the NCDA.

|  | No. of NCDA morbidities (excluding non- Charlson conditions*) | | | |
| --- | --- | --- | --- | --- |
| Charlson Comorbidity Index score | None | 1 condition | 2 conditions | 3+ conditions |
| Charlson score = 0 (no conditions) | 3841 (46%) | 157 (10%) | 68 (8%) | 25 (3%) |
| Charlson score = 1 | 3038 (36%) | 639 (39%) | 284 (32%) | 179 (23%) |
| Charlson score = 2 | 1206 (14%) | 529 (33%) | 337 (38%) | 275 (35%) |
| Charlson score = 3+ | 346 (4%) | 293 (18%) | 187 (21%) | 312 (39%) |

*excluding hypertension, physical disability, and severe mental illness which are not conditions included in the Charlson score

## S4 Emergency referrals including self-referral to A&E

We examined referral type including patient self-referrals to A&E in the emergency referral category (under orange headers). The direction and magnitude of associations were highly comparable to those we present in the main findings (under blue headers).

|  |  | **Referral type** | | |  |  |  |
| --- | --- | --- | --- | --- | --- | --- | --- |
|  |  | **2WW** | **Non-2WW** | **Emergency** | **Crude OR for Emergency referral** | **Adj OR* for Emergency referral** | **Adj OR** for Emergency referral** |
|  | Total N^ | N (%) | N (%) | N (%) | OR (95% CI) | OR (95% CI) | OR (95% CI) |
| Total | 11278 | 7785 (69%) | 1928 (17%) | 1565 (14%) | - | - | - |
| No. of NCDA morbidities | |  |  |  | **<0.001‡** | **<0.001‡** | **<0.001‡** |
| 0 morbidities | 2783 | 2001 (72%) | 518 (19%) | 264 (9%) | Ref | Ref | Ref |
| 1 morbidity | 3508 | 2436 (69%) | 626 (18%) | 446 (13%) | **1.39 (1.18–1.63)** | **1.23 (1.04–1.45)** | 1.15 (0.96–1.37) |
| 2 morbidities | 2712 | 1873 (69%) | 419 (15%) | 420 (15%) | **1.75 (1.48–2.06)** | **1.40 (1.17–1.67)** | **1.33 (1.10–1.61)** |
| 3+ morbidities | 2275 | 1475 (65%) | 365 (16%) | 435 (19%) | **2.26 (1.91–2.66)** | **1.67 (1.39–2.01)** | **1.56 (1.28–1.89)** |
| Charlson score |  |  |  |  | **<0.001‡** | **<0.001‡** | **<0.001‡** |
| 0 morbidities | 8156 | 5796 (71%) | 1378 (17%) | 982 (12%) | Ref | Ref | Ref |
| 1 morbidity | 1560 | 1005 (64%) | 282 (18%) | 273 (18%) | **1.55 (1.34–1.79)** | **1.38 (1.19–1.60)** | **1.32 (1.13–1.56)** |
| 2 morbidities | 825 | 535 (65%) | 150 (18%) | 140 (17%) | **1.49 (1.23–1.81)** | **1.29 (1.06–1.57)** | 1.18 (0.96–1.46) |
| 3+ morbidities | 737 | 449 (61%) | 118 (16%) | 170 (23%) | **2.19 (1.82–2.63)** | **1.75 (1.45–2.12)** | **1.57 (1.28–1.93)** |

^2WW = two-week-wait urgent referrals for suspected cancer; non-2WW includes routine, urgent non-cancer, and private referrals; emergency includes emergency referrals and patient self-referrals to A&E; 438 (4%) individuals whose referrals were categorised as “other”, “screen-detected”, and “not known” were excluded.

‡Wald test for overall significance

*Model adjusted for number of morbidities, sex, age, ethnicity, and Index of Multiple Deprivation (IMD)

**Model adjusted for number of morbidities, sex, age, ethnicity, IMD, and cancer. No melanoma patients were referred as an emergency and so these individuals (n=665) were excluded from the model

## S5 Analysis by morbidity combination

We considered patients with each of the 11 specific morbidities in our data using the following combinations:

- No morbidities at all (n=2872, 25% of the study population);
- No “specific morbidity”;
- The “specific morbidity” only;
- The “specific morbidity” and one or more other morbidities.

The relative frequencies are described below; given the small numbers of patients in certain strata, we focused on the **five most common morbidities** (excluding “Other” morbidity). Analyses presented subsequently are presented by morbidity combinations for each of the five morbidities.

|  | No “specific morbidity” | | The “specific morbidity” only | | The “specific morbidity” and 1+ morbidities | |
| --- | --- | --- | --- | --- | --- | --- |
|  | N | % (95% CI) | N | % (95% CI) | N | % (95% CI) |
| Hypertension | 4302 | 37% (36–38%) | 1091 | 9% (9–10%) | 3451 | 29% (29–30%) |
| CVD | 6490 | 55% (54–56%) | 370 | 3% (3–3%) | 1984 | 17% (16–18%) |
| Other morbidity | 6611 | 56% (56–57%) | 642 | 5% (5–6%) | 1591 | 14% (13–14%) |
| MSK disease | 6731 | 57% (57–58%) | 428 | 4% (3–4%) | 1685 | 14% (14–15%) |
| Respiratory | 7147 | 61% (60–62%) | 379 | 3% (3–4%) | 1318 | 11% (11–12%) |
| Diabetes | 6978 | 60% (59–60%) | 256 | 2% (2–2%) | 1610 | 14% (13–14%) |
| Previous cancer | 7750 | 66% (65–67%) | 244 | 2% (2–2%) | 850 | 7% (7–8%) |
| Stroke | 8075 | 69% (68–70%) | 59 | 1% (0–1%) | 710 | 6% (6–7%) |
| Cognitive impairment | 8387 | 72% (71–72%) | 65 | 1% (0–1%) | 392 | 3% (3–4%) |
| Severe mental illness | 8561 | 73% (72–74%) | 86 | 1% (1–1%) | 197 | 2% (1–2%) |
| Physical disability | 8677 | 74% (73–75%) | 13 | 0.1% (0.1–0.2%) | 154 | 1% (1–2%) |

CBD: cerebrovascular disease; CVD: cardiovascular disease; MSK disease: arthritis/ musculo-skeletal disease; Respiratory disease: chronic respiratory illness

### S5.1 Morbidities and primary care interval, by morbidity combination

|  |  | Centiles of interest | | | | | PCI <=28 days | PCI >28 days | Crude OR for PCI >28 days | Adjusted OR* for PCI >28 days | Adjusted OR** for PCI >28 days |
| --- | --- | --- | --- | --- | --- | --- | --- | --- | --- | --- | --- |
|  | N^ | 10^th^ | 25^th^ | 50^th^ | 75^th^ | 90^th^ | N (%) | N (%) | OR (95% CI) | OR (95% CI) | OR (95% CI) |
| Hypertension | | **<0.001†** | | | | |  |  | **0.014**‡ | **0.034**‡ | 0.321‡ |
| Nothing at all | 2342 | 0 | 0 | 3 | 23 | 77 | 1824 (78%) | 518 (22%) | Ref | Ref | Ref |
| No hypertension | 3421 | 0 | 0 | 6 | 30 | 84 | 2543 (74%) | 878 (26%) | **1.22 (1.07–1.38)** | **1.20 (1.05–1.36)** | 1.12 (0.98–1.29) |
| Hypertension only | 886 | 0 | 0 | 6 | 27 | 70 | 677 (76%) | 209 (24%) | 1.09 (0.91–1.31) | 1.07 (0.88–1.29) | 1.07 (0.88–1.30) |
| Hyp & 1+ morbidity | 2753 | 0 | 0 | 4 | 26 | 69 | 2112 (77%) | 641 (23%) | 1.07 (0.94–1.22) | 1.06 (0.92–1.23) | 1.03 (0.89–1.20) |
| CVD | | **<0.001†** | | | | |  |  | 0.108‡ | 0.188‡ | 0.440‡ |
| Nothing at all | 2342 | 0 | 0 | 3 | 23 | 77 | 1824 (78%) | 518 (22%) | Ref | Ref | Ref |
| No CVD | 5217 | 0 | 0 | 5 | 28 | 74 | 3953 (76%) | 1264 (24%) | **1.13 (>1.00–1.26)** | 1.13 (0.99–1.28) | 1.08 (0.95–1.23) |
| CVD only | 307 | 0 | 0 | 7 | 28 | 108 | 231 (75%) | 76 (25%) | 1.16 (0.88–1.53) | 1.10 (0.83–1.46) | 1.05 (0.79–1.41) |
| CVD & 1+ morbidity | 1536 | 0 | 0 | 6 | 29 | 77 | 1148 (75%) | 388 (25%) | **1.19 (1.02–1.38)** | **1.19 (1.01–1.41)** | 1.15 (0.97–1.37) |
| Arthritis/MSK disease | | **<0.001†** | | | | |  |  | 0.099‡ | 0.088‡ | 0.231‡ |
| Nothing at all | 2342 | 0 | 0 | 3 | 23 | 77 | 1824 (78%) | 518 (22%) | Ref | Ref | Ref |
| No MSK | 5384 | 0 | 0 | 5.5 | 28 | 74 | 4076 (76%) | 1308 (24%) | **1.13 (1.01–1.27)** | 1.12 (0.98–1.27) | 1.06 (0.94–1.21) |
| MSK only | 343 | 0 | 0 | 3 | 26 | 74 | 262 (76%) | 81 (24%) | 1.09 (0.83–1.42) | 1.10 (0.84–1.44) | 1.13 (0.85–1.50) |
| MSK & 1+ morbidity | 1333 | 0 | 0 | 5 | 29 | 85 | 994 (75%) | 339 (25%) | **1.20 (1.03–1.41)** | **1.25 (1.05–1.48)** | **1.20 (>1.00–1.43)** |
| Respiratory illness | | **<0.001†** | | | | |  |  | **0.004** | **0.011‡** | 0.176‡ |
| Nothing at all | 2342 | 0 | 0 | 3 | 23 | 77 | 1824 (78%) | 518 (22%) | Ref | Ref | Ref |
| No resp illness | 5738 | 0 | 0 | 5 | 27 | 75 | 4369 (76%) | 1369 (24%) | 1.10 (0.98–1.24) | 1.10 (0.97–1.25) | 1.08 (0.95–1.23) |
| Resp illness only | 308 | 0 | 0 | 7 | 27.5 | 86 | 232 (75%) | 76 (25%) | 1.15 (0.87–1.52) | 1.15 (0.87–1.52) | 0.92 (0.69–1.23) |
| Resp illness & 1+ morbidity | 1014 | 0 | 0 | 7 | 32 | 77 | 731 (72%) | 283 (28%) | **1.36 (1.15–1.61)** | **1.35 (1.13–1.62)** | 1.20 (0.99–1.44) |
| Diabetes | | **0.001†** | | | | |  |  | 0.107‡ | 0.195‡ | 0.567‡ |
| Nothing at all | 2342 | 0 | 0 | 3 | 23 | 77 | 1824 (78%) | 518 (22%) | Ref | Ref | Ref |
| No Diabetes | 5580 | 0 | 0 | 6 | 28 | 76 | 4213 (76%) | 1367 (24%) | **1.14 (1.02–1.28)** | **1.14 (1.01–1.29)** | 1.10 (0.96–1.25) |
| Diabetes only | 198 | 0 | 0 | 6.5 | 31 | 92 | 145 (73%) | 53 (27%) | 1.29 (0.93–1.79) | 1.20 (0.86–1.68) | 1.09 (0.77–1.53) |
| Diabetes & 1+ morbidity | 1282 | 0 | 0 | 4 | 28 | 71 | 974 (76%) | 308 (24%) | 1.11 (0.95–1.31) | 1.09 (0.91–1.29) | 1.05 (0.88–1.25) |

^Total n=9402, 80% of patients had complete information on the PCI

†Kruskal-Wallis test p-value at 50^th^ centile

‡Wald test for overall significance

*Model adjusted for number of morbidities, sex, age, ethnicity, and IMD

**Model adjusted for number of morbidities, sex, age, ethnicity, IMD, and cancer

### S5.2 Morbidities and diagnostic interval, by morbidity combination

|  |  | Centiles of interest | | | | | DI <=60 days | DI >60 days | Crude OR for DI >60 days | Adjusted OR* for DI >60 days | Adjusted OR** for DI >60 days |
| --- | --- | --- | --- | --- | --- | --- | --- | --- | --- | --- | --- |
|  | N^ | 10^th^ | 25^th^ | 50^th^ | 75^th^ | 90^th^ | N (%) | N (%) | OR (95% CI) | OR (95% CI) | OR (95% CI) |
| Hypertension | | **<0.001†** | | | | |  |  | **0.005‡** | **0.017‡** | 0.106‡ |
| Nothing at all | 2476 | 9 | 15 | 38 | 82 | 177 | 1615 (65%) | 861 (35%) | Ref | Ref | Ref |
| No hypertension | 3753 | 10 | 19 | 44 | 94 | 196 | 2284 (61%) | 1469 (39%) | **1.21 (1.09–1.34)** | **1.19 (1.06–1.33)** | **1.15 (1.02–1.29)** |
| Hypertension only | 961 | 9 | 17 | 42 | 89 | 193 | 613 (64%) | 348 (36%) | 1.06 (0.91–1.24) | 1.04 (0.88–1.22) | 1.03 (0.87–1.21) |
| Hyp & 1+ morbidity | 3046 | 10 | 19 | 43 | 91 | 191 | 1893 (62%) | 1153 (38%) | **1.14 (1.02–1.28)** | **1.13 (>1.00–1.28)** | 1.11 (0.97–1.26) |
| CVD | | **0.001†** | | | | |  |  | **0.002‡** | 0.032‡ | 0.126‡ |
| Nothing at all | 2476 | 9 | 15 | 38 | 82 | 177 | 1615 (65%) | 861 (35%) | Ref | Ref | Ref |
| No CVD | 5713 | 10 | 18 | 43 | 91 | 191 | 3554 (62%) | 2159 (38%) | **1.14 (1.03–1.26)** | **1.14 (1.02–1.26)** | 1.10 (0.99–1.23) |
| CVD only | 325 | 10 | 23 | 50 | 109 | 220 | 183 (56%) | 142 (44%) | **1.46 (1.15–1.84)** | **1.34 (1.05–1.70)** | 1.28 (<1.00–1.64) |
| CVD & 1+ morbidity | 1722 | 10 | 20 | 44 | 95 | 196 | 1053 (61%) | 669 (39%) | **1.19 (1.05–1.35)** | **1.17 (1.02–1.35)** | 1.15 (0.99–1.33) |
| Arthritis/MSK disease | | **<0.001†** | | | | |  |  | **0.014** | 0.064‡ | 0.212‡ |
| Nothing at all | 2476 | 9 | 15 | 38 | 82 | 177 | 1615 (65%) | 861 (35%) | Ref | Ref | Ref |
| No MSK | 5902 | 10 | 19 | 44 | 92 | 192 | 3629 (61%) | 2273 (39%) | **1.17 (1.07–1.30)** | **1.15 (1.03–1.27)** | 1.11 (0.99–1.24) |
| MSK only | 370 | 11 | 18 | 39 | 87 | 178 | 235 (64%) | 135 (36%) | 1.08 (0.86–1.35) | 1.10 (0.87–1.39) | 1.12 (0.87–1.42) |
| MSK & 1+ morbidity | 1488 | 10 | 19 | 43 | 95 | 209 | 926 (62%) | 562 (38%) | 1.14 (<1.00–1.30) | **1.18 (1.02–1.37)** | 1.16 (<1.00–1.35) |
| Respiratory illness | | **<0.001†** | | | | |  |  | **0.007‡** | 0.039‡ | 0.125‡ |
| Nothing at all | 2476 | 9 | 15 | 38 | 82 | 177 | 1615 (65%) | 861 (35%) | Ref | Ref | Ref |
| No resp illness | 6273 | 10 | 18 | 43 | 92 | 194 | 3890 (62%) | 2383 (38%) | **1.15 (1.04–1.27)** | **1.14 (1.02–1.27)** | 1.12 (<1.00–1.25) |
| Resp illness only | 338 | 11 | 25 | 46.5 | 87 | 162 | 213 (63%) | 125 (37%) | 1.10 (0.87–1.39) | 1.09 (0.86–1.38) | 0.99 (0.77–1.27) |
| Resp illness & 1+ morbidity | 1149 | 11 | 21 | 48 | 97 | 209 | 687 (60%) | 462 (40%) | **1.26 (1.09–1.46)** | **1.23 (1.06–1.44)** | **1.18 (>1.00–1.38)** |
| Diabetes | | **<0.001†** | | | | |  |  | **0.019‡** | 0.063‡ | 0.195‡ |
| Nothing at all | 2476 | 9 | 15 | 38 | 82 | 177 | 1615 (65%) | 861 (35%) | Ref | Ref | Ref |
| No Diabetes | 6139 | 10 | 19 | 43 | 92 | 196 | 3792 (62%) | 2347 (38%) | **1.16 (1.05–1.28)** | **1.16 (1.04–1.29)** | **1.13 (1.01–1.26)** |
| Diabetes only | 208 | 10 | 18.5 | 46.5 | 105.5 | 185 | 126 (61%) | 82 (39%) | 1.22 (0.91–1.63) | 1.12 (0.84–1.50) | 1.04 (0.77–1.41) |
| Diabetes & 1+ morbidity | 1413 | 10 | 18 | 44 | 92 | 190 | 872 (62%) | 541 (38%) | **1.16 (1.02–1.33)** | 1.11 (0.96–1.28) | 1.08 (0.93–1.26) |

^Total n=10236, 87% of patients had complete information on the DI

†Kruskal-Wallis test p-value at 50th centile

‡Wald test for overall significance

*Model adjusted for number of morbidities, sex, age, ethnicity, and IMD

**Model adjusted for number of morbidities, sex, age, ethnicity, IMD, and cancer

### S5.3 Morbidities and pre-referral consultations, by morbidity combination

|  | **N**^ | **3+ consultations**  **N (%)** | **Crude OR for 3+ consultations OR (95% CI)** | **Adjusted OR* for 3+ consultations**  **OR (95% CI)** | **Adjusted OR** for 3+ consultations**  **OR (95% CI)** |
| --- | --- | --- | --- | --- | --- |
| Hypertension |  |  | **<0.001‡** | **0.004‡** | 0.340‡ |
| Nothing at all | 2839 | 599 (21%) | Ref | Ref | Ref |
| No hypertension | 4198 | 1121 (27%) | **1.36 (1.22–1.53)** | **1.20 (1.06–1.35)** | 1.08 (0.95–1.23) |
| Hypertension only | 1075 | 242 (23%) | 1.09 (0.92–1.29) | 0.95 (0.80–1.13) | 0.94 (0.79–1.13) |
| Hyp & 1+ morbidity | 3361 | 875 (26%) | **1.32 (1.17–1.48)** | 1.09 (0.96–1.25) | 1.05 (0.91–1.20) |
| CVD |  |  | **<0.001‡** | 0.089‡ | 0.426‡ |
| Nothing at all | 2839 | 599 (21%) | Ref | Ref | Ref |
| No CVD | 6340 | 1590 (25%) | **1.25 (1.13–1.39)** | 1.11 (0.99–1.24) | 1.03 (0.92–1.17) |
| CVD only | 368 | 105 (29%) | **1.49 (1.17–1.90)** | 1.22 (0.95–1.56) | 1.16 (0.90–1.50) |
| CVD & 1+ morbidity | 1926 | 543 (28%) | **1.47 (1.28–1.68)** | **1.20 (1.03–1.39)** | 1.11 (0.96–1.30) |
| Arthritis/MSK disease |  |  | **<0.001‡** | **0.014‡** | 0.079‡ |
| Nothing at all | 2839 | 599 (21%) | Ref | Ref | Ref |
| No MSK | 6573 | 1691 (26%) | **1.30 (1.17–1.44)** | **1.12 (>1.00–1.26)** | 1.04 (0.92–1.17) |
| MSK only | 418 | 88 (21%) | 1.00 (0.78–1.28) | 0.91 (0.71–1.17) | 0.90 (0.69–1.17) |
| MSK & 1+ morbidity | 1643 | 459 (28%) | **1.45 (1.26–1.67)** | **1.24 (1.07–1.44)** | **1.19 (1.01–1.39)** |
| Respiratory illness |  |  | **<0.001‡** | **0.001‡** | 0.692‡ |
| Nothing at all | 2839 | 599 (21%) | Ref | Ref | Ref |
| No resp illness | 6986 | 1756 (25%) | **1.26 (1.13–1.39)** | 1.08 (0.97–1.21) | 1.04 (0.92–1.17) |
| Resp illness only | 369 | 104 (28%) | **1.47 (1.15–1.87)** | **1.35 (1.06–1.73)** | 1.07 (0.83–1.39) |
| Resp illness & 1+ morbidity | 1279 | 378 (30%) | **1.57 (1.35–1.82)** | **1.33 (1.13–1.56)** | 1.11 (0.94–1.31) |
| Diabetes |  |  | **<0.001‡** | **0.029‡** | 0.130‡ |
| Nothing at all | 2839 | 599 (21%) | Ref | Ref | Ref |
| No Diabetes | 6819 | 1794 (26%) | **1.34 (1.20–1.48)** | **1.15 (1.03–1.29)** | 1.08 (0.96–1.22) |
| Diabetes only | 248 | 64 (26%) | 1.30 (0.97–1.75) | 1.16 (0.86–1.57) | 0.97 (0.71–1.33) |
| Diabetes & 1+ morbidity | 1567 | 380 (24%) | **1.20 (1.03–1.39)** | 1.00 (0.86–1.17) | 0.93 (0.79–1.10) |

^Total n=11473, 98% of patients had complete information on number of pre-referral consultations

‡Wald test for overall significance

*Model adjusted for number of morbidities, sex, age, ethnicity, and IMD

**Model adjusted for number of morbidities, sex, age, ethnicity, IMD, and cancer

### S5.4 Morbidities and investigations, by morbidity combination

|  | **N^** | **1+ Investigations**  **N (%)** | **Crude OR for 1+ Investigations**  **OR (95% CI)** | **Adjusted OR* for 1+ Investigations**  **OR (95% CI)** | **Adjusted OR** for 1+ Investigations**  **OR (95% CI)** |
| --- | --- | --- | --- | --- | --- |
| Hypertension |  |  | **<0.001‡** | 0.465‡ | 0.373‡ |
| Nothing at all | 2762 | 1544 (56%) | Ref | Ref | Ref |
| No hypertension | 4112 | 2544 (62%) | **1.28 (1.16–1.41)** | 1.02 (0.92–1.14) | 0.90 (0.79–1.02) |
| Hypertension only | 1048 | 629 (60%) | **1.18 (1.02–1.37)** | 0.92 (0.79–1.08) | 0.92 (0.76–1.12) |
| Hyp & 1+ morbidity | 3308 | 2060 (62%) | **1.30 (1.17–1.44)** | 0.96 (0.85–1.09) | 0.89 (0.77–1.03) |
| CVD |  |  | **<0.001‡** | 0.931‡ | 0.369‡ |
| Nothing at all | 2762 | 1544 (56%) | Ref | Ref | Ref |
| No CVD | 6222 | 3787 (61%) | **1.23 (1.12–1.34)** | 1.00 (0.90–1.10) | 0.90 (0.80–1.02) |
| CVD only | 352 | 234 (66%) | **1.56 (1.24–1.98)** | 0.98 (0.76–1.25) | 0.91 (0.69–1.20) |
| CVD & 1+ morbidity | 1894 | 1212 (64%) | **1.40 (1.24–1.58)** | 0.96 (0.84–1.10) | 0.88 (0.74–1.03) |
| Arthritis/MSK disease |  |  | **<0.001‡** | 0.970‡ | 0.343‡ |
| Nothing at all | 2762 | 1544 (56%) | Ref | Ref | Ref |
| No MSK | 6448 | 4008 (62%) | **1.30 (1.18–1.42)** | 0.99 (0.89–1.09) | 0.89 (0.79–1.01) |
| MSK only | 412 | 240 (58%) | 1.10 (0.89–1.36) | 0.97 (0.78–1.20) | 0.90 (0.69–1.19) |
| MSK & 1+ morbidity | 1608 | 985 (61%) | **1.25 (1.10–1.41)** | 1.01 (0.88–1.16) | 0.93 (0.79–1.10) |
| Respiratory illness |  |  | **<0.001‡** | **0.001‡** | 0.078‡ |
| Nothing at all | 2762 | 1544 (56%) | Ref | Ref | Ref |
| No resp illness | 6836 | 4157 (61%) | **1.22 (1.12–1.34)** | 0.95 (0.86–1.05) | 0.89 (0.79–1.01) |
| Resp illness only | 365 | 248 (68%) | **1.67 (1.33–2.11)** | **1.48 (1.16–1.89)** | 1.15 (0.86–1.55) |
| Resp illness & 1+ morbidity | 1267 | 828 (65%) | **1.49 (1.30–1.71)** | 1.08 (0.93–1.26) | 0.85 (0.71–1.02) |
| Diabetes |  |  | **<0.001‡** | 0.664‡ | 0.324‡ |
| Nothing at all | 2762 | 1544 (56%) | Ref | Ref | Ref |
| No Diabetes | 6691 | 4108 (61%) | **1.25 (1.15–1.37)** | 0.99 (0.90–1.10) | 0.91 (0.80–1.03) |
| Diabetes only | 245 | 161 (66%) | **1.51 (1.15–1.99)** | 1.12 (0.84–1.48) | 0.93 (0.67–1.29) |
| Diabetes & 1+ morbidity | 1532 | 964 (63%) | **1.34 (1.18–1.52)** | 0.94 (0.82–1.09) | 0.86 (0.73–1.02) |

^Total n=11230, 96% of patients had complete information on investigations

‡Wald test for overall significance

*Model adjusted for number of morbidities, sex, age, ethnicity, and IMD

**Model adjusted for number of morbidities, sex, age, ethnicity, IMD, and cancer

### S5.5 Morbidities and emergency referral, by morbidity combination

|  |  | **Referral type^** | | |  |  |  |
| --- | --- | --- | --- | --- | --- | --- | --- |
|  |  | **2WW** | **Non-2WW** | **Emergency** | **Crude OR for Emergency referral** | **Adj OR* for Emergency referral** | **Adj OR** for Emergency referral** |
|  | Total N | N (%) | N (%) | N (%) | OR (95% CI) | OR (95% CI) | OR (95% CI) |
| Hypertension |  |  |  |  | **<0.001‡** | **<0.001‡** | **0.004‡** |
| Nothing at all | 2686 | 2001 (74%) | 518 (19%) | 167 (6%) | Ref | Ref | Ref |
| No hypertension | 3905 | 2778 (71%) | 718 (18%) | 409 (10%) | **1.76 (1.46–2.13)** | **1.47 (1.20–1.79)** | **1.30 (1.06–1.60)** |
| Hypertension only | 1012 | 760 (75%) | 185 (18%) | 67 (7%) | 1.07 (0.80–1.43) | 0.90 (0.67–1.22) | 0.92 (0.67–1.26) |
| Hyp & 1+ morbidity | 3100 | 2246 (72%) | 507 (16%) | 347 (11%) | **1.90 (1.57–2.30)** | **1.40 (1.13–1.74)** | **1.37 (1.10–1.72)** |
| CVD |  |  |  |  | **<0.001‡** | **0.006‡** | 0.055‡ |
| Nothing at all | 2686 | 2001 (74%) | 518 (19%) | 167 (6%) | Ref | Ref | Ref |
| No CVD | 5904 | 4293 (73%) | 1035 (18%) | 576 (10%) | **1.63 (1.36–1.95)** | **1.35 (1.12–1.64)** | **1.26 (1.03–1.54)** |
| CVD only | 346 | 246 (71%) | 69 (20%) | 31 (9%) | 1.48 (0.99–2.22) | 1.18 (0.78–1.79) | 1.09 (0.71–1.68) |
| CVD & 1+ morbidity | 1767 | 1245 (70%) | 306 (17%) | 216 (12%) | **2.10 (1.70–2.60)** | **1.49 (1.17–1.88)** | **1.39 (1.08–1.77)** |
| Arthritis/MSK disease |  |  |  |  | **<0.001‡** | **0.002‡** | **0.039‡** |
| Nothing at all | 2686 | 2001 (74%) | 518 (19%) | 167 (6%) | Ref | Ref | Ref |
| No MSK | 6098 | 4372 (72%) | 1098 (18%) | 628 (10%) | **1.73 (1.45–2.07)** | **1.41 (1.16–1.71)** | **1.29 (1.06–1.58)** |
| MSK only | 398 | 301 (76%) | 69 (17%) | 28 (7%) | 1.14 (0.75–1.73) | 0.96 (0.63–1.47) | 0.93 (0.59–1.45) |
| MSK & 1+ morbidity | 1521 | 1111 (73%) | 243 (16%) | 167 (11%) | **1.86 (1.49–2.33)** | **1.33 (1.04–1.70)** | **1.30 (1.01–1.69)** |
| Respiratory illness |  |  |  |  | **<0.001‡** | **<0.001‡** | **0.004‡** |
| Nothing at all | 2686 | 2001 (74%) | 518 (19%) | 167 (6%) | Ref | Ref | Ref |
| No resp illness | 6491 | 4698 (72%) | 1150 (18%) | 643 (10%) | **1.66 (1.39–1.98)** | **1.32 (1.09–1.60)** | **1.27 (1.04–1.56)** |
| Resp illness only | 344 | 263 (76%) | 56 (16%) | 25 (7%) | 1.18 (0.76–1.83) | 1.07 (0.69–1.67) | 0.81 (0.51–1.28) |
| Resp illness & 1+ morbidity | 1182 | 823 (70%) | 204 (17%) | 155 (13%) | **2.28 (1.81–2.87)** | **1.81 (1.42–2.32)** | **1.49 (1.15–1.93)** |
| Diabetes |  |  |  |  | **<0.001‡** | **0.003‡** | 0.084‡ |
| Nothing at all | 2686 | 2001 (74%) | 518 (19%) | 167 (6%) | Ref | Ref | Ref |
| No Diabetes | 6324 | 4589 (73%) | 1102 (17%) | 633 (10%) | **1.68 (1.41–2.00)** | **1.32 (1.09–1.61)** | **1.26 (1.03–1.54)** |
| Diabetes only | 240 | 175 (73%) | 41 (17%) | 24 (10%) | **1.68 (1.07–2.63)** | **1.65 (1.04–2.59)** | 1.21 (0.75–1.95) |
| Diabetes & 1+ morbidity | 1453 | 1020 (70%) | 267 (18%) | 166 (11%) | **1.95 (1.55–2.44)** | **1.54 (1.21–1.96)** | **1.37 (1.06–1.77)** |

^Total n=10703. 2WW = two-week-wait urgent referrals for suspected cancer; non-2WW includes routine, urgent non-cancer, and private referrals; emergency includes emergency referrals; 1013 (9%) individuals whose referrals were categorised as patient self-referrals to A&E, “other”, “screen-detected”, and “not known” were excluded.

*Model adjusted for number of morbidities, sex, age, ethnicity, and IMD

**Model adjusted for number of morbidities, sex, age, ethnicity, IMD, and cancer. No melanoma patients were referred as an emergency and so these individuals are excluded from the model

## S6 Analyses by cancer diagnostic difficulty

Cancer were grouped by diagnostic difficulty based on their symptom signatures into three categories:

| Cancer category based on (Koo *et al*, 2017) | Cancers |
| --- | --- |
| “Easy”: Low diagnostic difficulty  Narrow symptom signature and high PPV | breast, bladder, melanoma, thyroid, testicular, vulval, cervical, endometrial |
| “Medium”: Medium diagnostic difficulty  Broad symptom signature and varying PPV | prostate, colorectal cancer, stomach, lung, oesophageal, oropharyngeal, ovarian, renal, prostate, pancreatic, gallbladder, laryngeal, liver, small intestinal |
| “Hard”: High diagnostic difficulty  Broad symptom signature and low PPV | Brain, leukaemia, lymphoma, myeloma, mesothelioma, CUP, other |

Most patients (57%) were diagnosed with cancers in group 2, with over a quarter of patients in the “easy to diagnose” group

|  |  | No. of morbidities | | | |
| --- | --- | --- | --- | --- | --- |
|  | N (% of total) | None | 1 | 2 | 3+ |
| Total | 11716 (100%) | 2872 (25%) | 3633 (31%) | 2819 (24%) | 2392 (20%) |
| Cancer category |  |  |  |  |  |
| Easy to diagnose | 3116 (27%) | 1011 (35%) | 916 (25%) | 674 (24%) | 515 (22%) |
| Medium | 6683 (57%) | 1400 (49%) | 2097 (58%) | 1692 (60%) | 1494 (62%) |
| Hard to diagnose | 1917 (16%) | 461 (16%) | 620 (17%) | 453 (16%) | 383 (16%) |

We examined associations between NCDA defined morbidity and the five diagnostic measures of interest within each of the above defined cancer patient populations.

### S6.1 Morbidities and primary care interval, by cancer diagnostic difficulty

|  |  | Centiles of interest | | | | | PCI >28 days | Crude OR for PCI >28 days | Adjusted OR* for PCI >28 days | Adjusted OR** for PCI >28 days |
| --- | --- | --- | --- | --- | --- | --- | --- | --- | --- | --- |
|  | N^ | 10^th^ | 25^th^ | 50^th^ | 75^th^ | 90^th^ | N (%) | OR (95% CI) | OR (95% CI) | OR (95% CI) |
| All patients | 9402 | 0 | 0 | 5 | 27 | 77 | 2246 (24%) | – | – | – |
| No. of NCDA morbidities | | **0.001†** | | | | |  | 0.073‡ | 0.102‡ | 0.369‡ |
| 0 morbidities | 2342 | 0 | 0 | 3 | 23 | 77 | 518 (22%) | Ref | Ref | Ref |
| 1 morbidity | 2922 | 0 | 0 | 5 | 28 | 82 | 716 (25%) | **1.14 (>1.00–1.30)** | 1.13 (0.99–1.29) | 1.08 (0.94–1.24) |
| 2 morbidities | 2274 | 0 | 0 | 5 | 27 | 66 | 539 (24%) | 1.09 (0.95–1.25) | 1.09 (0.94–1.27) | 1.06 (0.91–1.24) |
| 3+ morbidities | 1864 | 0 | 0 | 5 | 29 | 84 | 473 (25%) | **1.20 (1.04–1.38)** | **1.21 (1.04–1.42)** | 1.16 (0.98–1.36) |
| Easy to diagnose | | 0.558† | | | | |  | 0.244‡ | 0.271‡ | 0.287‡ |
| 0 morbidities | 852 | 0 | 0 | 0 | 3 | 31 | 93 (11%) | Ref | Ref | Ref |
| 1 morbidity | 769 | 0 | 0 | 0 | 3 | 32 | 83 (11%) | 0.99 (0.72–1.35) | 1.06 (0.76–1.48) | 1.02 (0.72–1.44) |
| 2 morbidities | 575 | 0 | 0 | 0 | 4 | 28 | 54 (9%) | 0.85 (0.59–1.20) | 0.93 (0.62–1.38) | 0.90 (0.60–1.36) |
| 3+ morbidities | 448 | 0 | 0 | 0 | 5.5 | 39 | 60 (13%) | 1.26 (0.89–1.79) | 1.36 (0.89–2.09) | 1.34 (0.87–2.07) |
| Medium | | 0.392† | | | | |  | 0.457‡ | 0.209‡ | 0.350‡ |
| 0 morbidities | 1142 | 0 | 0 | 10 | 35 | 103 | 321 (28%) | Ref | Ref | Ref |
| 1 morbidity | 1684 | 0 | 0 | 10 | 36 | 103 | 500 (30%) | 1.08 (0.91–1.28) | 1.15 (0.97–1.36) | 1.13 (0.95–1.34) |
| 2 morbidities | 1364 | 0 | 0 | 9 | 33 | 74 | 371 (27%) | 0.96 (0.80–1.14) | 1.06 (0.88–1.28) | 1.02 (0.85–1.24) |
| 3+ morbidities | 1128 | 0 | 0 | 8 | 34 | 94 | 329 (29%) | 1.05 (0.88–1.26) | 1.20 (0.99–1.47) | 1.14 (0.93–1.39) |
| Hard to diagnose | | 0.892† | | | | |  | 0.356‡ | 0.217‡ | 0.178‡ |
| 0 morbidities | 348 | 0 | 0 | 7.5 | 39.5 | 92 | 104 (30%) | Ref | Ref | Ref |
| 1 morbidity | 469 | 0 | 0 | 9 | 34 | 90 | 133 (28%) | 0.93 (0.68–1.26) | 1.01 (0.73–1.39) | 0.96 (0.69–1.32) |
| 2 morbidities | 335 | 0 | 0 | 10 | 39 | 78 | 114 (34%) | 1.21 (0.88–1.67) | 1.37 (0.96–1.95) | 1.35 (0.94–1.92) |
| 3+ morbidities | 288 | 0 | 0 | 10 | 35 | 93 | 84 (29%) | 0.97 (0.69–1.36) | 1.10 (0.75–1.60) | 1.07 (0.73–1.57) |

^Total n=9402, 80% of patients had complete information on the PCI

†Kruskal-Wallis test p-value at 50^th^ centile

‡Wald test for overall significance

*Model adjusted for number of morbidities, sex, age, ethnicity, and IMD

**Model adjusted for number of morbidities, sex, age, ethnicity, IMD, and cancer (to account for potential cancer case-mix differences within each group of diagnostic difficulty)

### S6.2 Morbidities and diagnostic interval, by cancer diagnostic difficulty

|  |  | Centiles of interest | | | | | DI >60 days | Crude OR for DI >60 days | Adjusted OR* for DI >60 days | Adjusted OR** for DI >60 days |
| --- | --- | --- | --- | --- | --- | --- | --- | --- | --- | --- |
|  | N^ | 10^th^ | 25^th^ | 50^th^ | 75^th^ | 90^th^ | N (%) | OR (95% CI) | OR (95% CI) | OR (95% CI) |
| All patients | 10236 | 10 | 18 | 42 | 91 | 190 | 3831 (37%) | - | - | - |
| No of morbidities | | **<0.001†** | | | | |  | **<0.001‡** | **0.001‡** | **0.004‡** |
| 0 morbidities | 2476 | 9 | 15 | 38 | 82 | 177 | 861 (35%) | Ref | Ref | Ref |
| 1 morbidity | 3171 | 10 | 19 | 44 | 93 | 195 | 1216 (38%) | 1.17 (1.05–1.30) | 1.15 (1.02–1.29) | 1.11 (0.98–1.25) |
| 2 morbidities | 2485 | 10 | 18 | 42 | 85 | 182 | 902 (36%) | 1.07 (0.95–1.20) | 1.06 (0.93–1.20) | 1.04 (0.91–1.19) |
| 3+ morbidities | 2104 | 10 | 20 | 47 | 100 | 209 | 852 (40%) | **1.28 (1.13–1.44)** | **1.29 (1.13–1.48)** | **1.26 (1.10–1.45)** |
| Easy to diagnose | | **0.002†** | | | | |  | **0.004‡** | **0.010‡** | **0.027‡** |
| 0 morbidities | 870 | 7 | 12 | 18 | 46 | 116 | 167 (19%) | Ref | Ref | Ref |
| 1 morbidity | 800 | 8 | 12 | 18 | 49 | 114.5 | 167 (21%) | 1.11 (0.87–1.41) | 1.14 (0.88–1.48) | 1.12 (0.85–1.49) |
| 2 morbidities | 591 | 9 | 13 | 19 | 44 | 92 | 99 (17%) | 0.85 (0.64–1.11) | 0.85 (0.62–1.16) | 0.82 (0.59–1.15) |
| 3+ morbidities | 466 | 8 | 13 | 24 | 62 | 137 | 119 (26%) | **1.44 (1.10–1.89)** | **1.41 (1.02–1.96)** | 1.36 (0.96–1.93) |
| Medium | | 0.278† | | | | |  | 0.406‡ | **0.020‡** | **0.020‡** |
| 0 morbidities | 1209 | 14 | 26 | 49 | 95 | 200 | 511 (42%) | Ref | Ref | Ref |
| 1 morbidity | 1843 | 13 | 27 | 53 | 107 | 218 | 814 (44%) | 1.08 (0.93–1.25) | **1.17 (1.01–1.36)** | **1.17 (>1.00–1.36)** |
| 2 morbidities | 1509 | 12 | 25 | 49 | 99 | 206 | 636 (42%) | 1.00 (0.85–1.16) | 1.14 (0.97–1.34) | 1.13 (0.96–1.33) |
| 3+ morbidities | 1298 | 11 | 24 | 52 | 110 | 225 | 580 (45%) | **1.10 (0.94–1.29)** | **1.31 (1.10–1.56)** | **1.31 (1.10–1.56)** |
| Hard to diagnose | | 0.947† | | | | |  | 0.895‡ | 0.906‡ | 0.878‡ |
| 0 morbidities | 397 | 8 | 22 | 56 | 107 | 215 | 183 (46%) | Ref | Ref | Ref |
| 1 morbidity | 528 | 8 | 23 | 53 | 112 | 212 | 235 (45%) | 0.94 (0.72–1.22) | 0.97 (0.74–1.28) | 0.96 (0.73–1.27) |
| 2 morbidities | 385 | 11 | 23 | 52 | 99 | 189 | 167 (43%) | 0.90 (0.68–1.19) | 0.97 (0.72–1.32) | 0.95 (0.70–1.30) |
| 3+ morbidities | 340 | 7.5 | 24 | 51 | 108.5 | 220.5 | 153 (45%) | 0.96 (0.72–1.28) | 1.07 (0.78–1.48) | 1.07 (0.77–1.48) |

^Total n=10236, 87% of patients had complete information on the DI

†Kruskal-Wallis test p-value at 50th centile

‡Wald test for overall significance

*Model adjusted for number of morbidities, sex, age, ethnicity, and IMD

**Model adjusted for number of morbidities, sex, age, ethnicity, IMD, and cancer (to account for potential cancer case-mix differences within each group of diagnostic difficulty)

### S6.3 Morbidities and pre-referral consultations, by cancer diagnostic difficulty

|  | **N**^ | **3+ consultations**  **N (%)** | **Crude OR for 3+ consultations OR (95% CI)** | **Adjusted OR* for 3+ consultations**  **OR (95% CI)** | **Adjusted OR** for 3+ consultations**  **OR (95% CI)** |
| --- | --- | --- | --- | --- | --- |
| All patients | 11473 | 2837 (25%) | - | - | - |
| No of morbidities |  |  | **<0.001‡** | **<0.001‡** | **0.010‡** |
| 0 morbidities | 2839 | 599 (21%) | Ref | Ref | Ref |
| 1 morbidity | 3554 | 880 (25%) | 1.23 (1.09–1.38) | 1.11 (0.98–1.25) | 1.03 (0.91–1.17) |
| 2 morbidities | 2760 | 675 (24%) | 1.21 (1.07–1.37) | 1.04 (0.91–1.19) | 0.98 (0.85–1.13) |
| 3+ morbidities | 2320 | 683 (29%) | **1.56 (1.37–1.77)** | **1.31 (1.14–1.51)** | **1.21 (1.05–1.40)** |
| Easy to diagnose |  |  | **0.010‡** | 0.232‡ | 0.341‡ |
| 0 morbidities | 1009 | 67 (7%) | Ref | Ref | Ref |
| 1 morbidity | 907 | 82 (9%) | 1.40 (<1.00–1.96) | 1.15 (0.80–1.65) | 1.07 (0.73–1.56) |
| 2 morbidities | 666 | 50 (8%) | 1.14 (0.78–1.67) | 0.82 (0.54–1.26) | 0.80 (0.51–1.24) |
| 3+ morbidities | 506 | 58 (11%) | **1.82 (1.26–2.63)** | 1.19 (0.77–1.85) | 1.15 (0.73–1.82) |
| Medium |  |  | **0.002‡** | **0.004‡** | **0.030‡** |
| 0 morbidities | 1377 | 389 (28%) | Ref | Ref | Ref |
| 1 morbidity | 2053 | 611 (30%) | 1.08 (0.93–1.25) | 1.08 (0.92–1.26) | 1.04 (0.89–1.21) |
| 2 morbidities | 1662 | 476 (29%) | 1.02 (0.87–1.19) | 1.03 (0.87–1.22) | 0.97 (0.81–1.15) |
| 3+ morbidities | 1443 | 492 (34%) | **1.31 (1.12–1.54)** | **1.32 (1.11–1.57)** | **1.21 (1.02–1.45)** |
| Hard to diagnose |  |  | 0.419‡ | 0.458‡ | 0.396‡ |
| 0 morbidities | 453 | 143 (32%) | Ref | Ref | Ref |
| 1 morbidity | 594 | 187 (31%) | 1.00 (0.77–1.30) | 1.01 (0.77–1.33) | 0.97 (0.73–1.28) |
| 2 morbidities | 432 | 149 (34%) | 1.14 (0.86–1.51) | 1.14 (0.84–1.54) | 1.14 (0.84–1.55) |
| 3+ morbidities | 371 | 133 (36%) | 1.21 (0.91–1.62) | 1.24 (0.90–1.72) | 1.22 (0.88–1.69) |

^Total n=11473, 98% of patients had complete information on number of pre-referral consultations

‡Wald test for overall significance

*Model adjusted for number of morbidities, sex, age, ethnicity, and IMD

**Model adjusted for number of morbidities, sex, age, ethnicity, IMD, and cancer (to account for potential cancer case-mix differences within each group of diagnostic difficulty)

### S6.4 Morbidities and investigations, by cancer diagnostic difficulty

|  | **N^** | **1+ Investigations**  **N (%)** | **Crude OR for 1+ Investigations**  **OR (95% CI)** | **Adjusted OR* for 1+ Investigations**  **OR (95% CI)** | **Adjusted OR** for 1+ Investigations**  **OR (95% CI)** |
| --- | --- | --- | --- | --- | --- |
| Total | 11230 | 6777 (60%) | - | - | - |
| No of morbidities |  |  | **<0.001‡** | 0.914‡ | 0.365‡ |
| 0 morbidities | 2762 | 1544 (56%) | Ref | Ref | Ref |
| 1 morbidity | 3487 | 2128 (61%) | 1.24 (1.12–1.37) | 1.01 (0.90–1.12) | 0.91 (0.80–1.04) |
| 2 morbidities | 2703 | 1678 (62%) | 1.29 (1.16–1.44) | 0.97 (0.86–1.10) | 0.89 (0.77–1.03) |
| 3+ morbidities | 2278 | 1427 (63%) | 1.32 (1.18–1.48) | 0.97 (0.85–1.11) | 0.88 (0.76–1.03) |
| Easy to diagnose |  |  | 0.088‡ | 0.724‡ | 0.893‡ |
| 0 morbidities | 947 | 180 (19%) | Ref | Ref | Ref |
| 1 morbidity | 865 | 185 (21%) | 1.16 (0.92–1.46) | 1.10 (0.86–1.41) | 1.03 (0.76–1.40) |
| 2 morbidities | 626 | 140 (22%) | 1.23 (0.96–1.57) | 1.10 (0.83–1.47) | 1.07 (0.75–1.51) |
| 3+ morbidities | 479 | 118 (25%) | 1.39 (1.07–1.81) | 1.20 (0.87–1.65) | 1.16 (0.79–1.69) |
| Medium |  |  | 0.324‡ | 0.392‡ | 0.273‡ |
| 0 morbidities | 1370 | 1048 (76%) | Ref | Ref | Ref |
| 1 morbidity | 2030 | 1529 (75%) | 0.94 (0.80–1.10) | 0.91 (0.77–1.08) | 0.91 (0.77–1.08) |
| 2 morbidities | 1643 | 1233 (75%) | 0.92 (0.78–1.09) | 0.91 (0.76–1.08) | 0.91 (0.76–1.08) |
| 3+ morbidities | 1441 | 1059 (73%) | 0.85 (0.72–1.01) | 0.85 (0.70–1.02) | 0.85 (0.70–1.02) |
| Hard to diagnose |  |  | 0.980‡ | 0.849‡ | 0.579‡ |
| 0 morbidities | 445 | 316 (71%) | Ref | Ref | Ref |
| 1 morbidity | 592 | 414 (70%) | 0.95 (0.72–1.24) | 0.90 (0.68–1.19) | 0.81 (0.61–1.09) |
| 2 morbidities | 434 | 305 (70%) | 0.97 (0.72–1.29) | 0.89 (0.65–1.22) | 0.85 (0.62–1.18) |
| 3+ morbidities | 358 | 250 (70%) | 0.94 (0.70–1.28) | 0.87 (0.63–1.22) | 0.86 (0.61–1.21) |

^Total n=11230, 96% of patients had complete information on investigations

*Model adjusted for number of morbidities, sex, age, ethnicity, and IMD

**Model adjusted for number of morbidities, sex, age, ethnicity, IMD, and cancer (to account for potential cancer case-mix differences within each group of diagnostic difficulty)

### S6.5 Morbidities and emergency referral, by cancer diagnostic difficulty

|  |  | **Referral type** | | |  |  |  |
| --- | --- | --- | --- | --- | --- | --- | --- |
|  |  | **2WW** | **Non-2WW** | **Emergency** | **Crude OR for Emergency referral** | **Adj OR* for Emergency referral** | **Adj OR** for Emergency referral** |
|  | Total N^ | N (%) | N (%) | N (%) | OR (95% CI) | OR (95% CI) | OR (95% CI) |
| Total | 10703 | 7785 (73%) | 1928 (18%) | 990 (9%) | - | - | - |
| No of morbidities |  |  |  |  | **<0.001‡** | **<0.001‡** | **<0.001‡** |
| 0 morbidities | 2686 | 2001 (74%) | 518 (19%) | 167 (6%) | Ref | Ref | Ref |
| 1 morbidity | 3346 | 2436 (73%) | 626 (19%) | 284 (8%) | 1.40 (1.15–1.71) | 1.24 (1.01–1.53) | 1.14 (0.92–1.42) |
| 2 morbidities | 2550 | 1873 (73%) | 419 (16%) | 258 (10%) | 1.70 (1.39–2.08) | 1.36 (1.09–1.69) | 1.28 (1.02–1.61) |
| 3+ morbidities | 2121 | 1475 (70%) | 365 (17%) | 281 (13%) | **2.30 (1.88–2.82)** | **1.72 (1.37–2.15)** | **1.60 (1.26–2.02)** |
| Easy to diagnose |  |  |  |  | **0.016‡** | 0.193‡ | 0.141‡ |
| 0 morbidities | 992 | 833 (84%) | 148 (15%) | 11 (1%) | Ref | Ref | Ref |
| 1 morbidity | 885 | 761 (86%) | 107 (12%) | 17 (2%) | 1.75 (0.81–3.75) | 1.39 (0.60–3.24) | 1.32 (0.56–3.11) |
| 2 morbidities | 656 | 586 (89%) | 63 (10%) | 7 (1%) | 0.96 (0.37–2.49) | 0.60 (0.20–1.75) | 0.56 (0.19–1.65) |
| 3+ morbidities | 491 | 426 (87%) | 49 (10%) | 16 (3%) | **3.00 (1.38–6.52)** | 1.50 (0.57–3.92) | 1.60 (0.62–4.17) |
| Medium |  |  |  |  | **<0.001‡** | **<0.001‡** | **0.001‡** |
| 0 morbidities | 1296 | 944 (73%) | 253 (20%) | 99 (8%) | Ref | Ref | Ref |
| 1 morbidity | 1928 | 1388 (72%) | 365 (19%) | 175 (9%) | 1.21 (0.93–1.56) | 1.16 (0.89–1.52) | 1.09 (0.83–1.44) |
| 2 morbidities | 1516 | 1080 (71%) | 248 (16%) | 188 (12%) | **1.71 (1.33–2.21)** | **1.57 (1.19–2.06)** | **1.39 (1.05–1.85)** |
| 3+ morbidities | 1304 | 877 (67%) | 228 (17%) | 199 (15%) | **2.18 (1.69–2.81)** | **1.88 (1.42–2.49)** | **1.63 (1.22–2.18)** |
| Hard to diagnose |  |  |  |  | 0.214‡ | 0.457‡ | 0.361‡ |
| 0 morbidities | 398 | 224 (56%) | 117 (29%) | 57 (14%) | Ref | Ref | Ref |
| 1 morbidity | 533 | 287 (54%) | 154 (29%) | 92 (17%) | 1.25 (0.87–1.79) | 1.21 (0.83–1.76) | 1.24 (0.84–1.83) |
| 2 morbidities | 378 | 207 (55%) | 108 (29%) | 63 (17%) | 1.20 (0.81–1.77) | 1.09 (0.72–1.67) | 1.20 (0.77–1.86) |
| 3+ morbidities | 326 | 172 (53%) | 88 (27%) | 66 (20%) | **1.52 (1.03–2.24)** | 1.38 (0.90–2.13) | 1.50 (0.96–2.34) |

^Total n=10703. 2WW = two-week-wait urgent referrals for suspected cancer; non-2WW includes routine, urgent non-cancer, and private referrals; emergency includes emergency referrals; 1013 (9%) individuals whose referrals were categorised as patient self-referrals to A&E, “other”, “screen-detected”, and “not known” were excluded.

*Model adjusted for number of morbidities, sex, age, ethnicity, and IMD

**Model adjusted for number of morbidities, sex, age, ethnicity, IMD, and cancer (to account for potential cancer case-mix differences within each group of diagnostic difficulty)

## S7 Lung and colorectal cancer-specific analyses

Several published examples of associations between morbidities and measures of the diagnostic process in primary care were in patients diagnosed with lung or colorectal cancer. Therefore, we examined the associations presented in the main findings restricted to each of these cancers. Two models (crude and adjusted) were run.

|  |  | No. of NCDA morbidities | | | |
| --- | --- | --- | --- | --- | --- |
|  | N | None | 1 | 2 | 3+ |
| Main study population | 11716 | 2872 (25%) | 3633 (31%) | 2819 (24%) | 2392 (20%) |
| Colorectal cancer | 1368 | 314 (23%) | 406 (30%) | 339 (25%) | 309 (23%) |
| Lung cancer | 1460 | 212 (15%) | 417 (29%) | 409 (28%) | 422 (29%) |

|  |  | Charlson score | | | |
| --- | --- | --- | --- | --- | --- |
|  | N | 0 | 1 | 2 | 3+ |
| Main study population | 11716 | 8431 (72%) | 1618 (14%) | 876 (7%) | 791 (7%) |
| Colorectal cancer | 1368 | 981 (72%) | 185 (14%) | 104 (8%) | 98 (7%) |
| Lung cancer | 1460 | 891 (61%) | 262 (18%) | 147 (10%) | 160 (11%) |

### S7.1 Morbidities and primary care interval, among patients diagnosed with lung and colorectal cancer only

|  |  | Centiles of interest | | | | | PCI >28 days | Crude OR for PCI >28 days | Adjusted OR* for PCI >28 days | Adjusted OR** for PCI >28 days |
| --- | --- | --- | --- | --- | --- | --- | --- | --- | --- | --- |
|  | N^ | 10^th^ | 25^th^ | 50^th^ | 75^th^ | 90^th^ | N (%) | OR (95% CI) | OR (95% CI) | OR (95% CI) |
| All patients | 9402 | 0 | 0 | 5 | 27 | 77 | 2246 (24%) | – | – | – |
| No of morbidities | | **0.001†** | | | | |  | 0.073‡ | 0.102‡ | 0.369‡ |
| 0 morbidities | 2342 | 0 | 0 | 3 | 23 | 77 | 518 (22%) | Ref | Ref | Ref |
| 1 morbidity | 2922 | 0 | 0 | 5 | 28 | 82 | 716 (25%) | **1.14 (>1.00–1.30)** | 1.13 (0.99–1.29) | 1.08 (0.94–1.24) |
| 2 morbidities | 2274 | 0 | 0 | 5 | 27 | 66 | 539 (24%) | 1.09 (0.95–1.25) | 1.09 (0.94–1.27) | 1.06 (0.91–1.24) |
| 3+ morbidities | 1864 | 0 | 0 | 5 | 29 | 84 | 473 (25%) | **1.20 (1.04–1.38)** | **1.21 (1.04–1.42)** | 1.16 (0.98–1.36) |
| Colorectal cancer | | 0.166† | | | | |  | 0.652‡ | 0.675‡ |  |
| 0 morbidities | 267 | 0 | 0 | 1 | 23 | 87 | 58 (22%) | Ref | Ref | – |
| 1 morbidity | 325 | 0 | 0 | 6 | 30 | 120 | 84 (26%) | 1.26 (0.86–1.84) | 1.25 (0.84–1.87) | – |
| 2 morbidities | 288 | 0 | 0 | 6 | 28 | 89 | 68 (24%) | 1.11 (0.75–1.66) | 1.14 (0.74–1.76) | – |
| 3+ morbidities | 237 | 0 | 0 | 3 | 22 | 122 | 53 (22%) | 1.04 (0.68–1.58) | 1.04 (0.65–1.66) | – |
| Lung cancer | | 0.603† | | | | |  | 0.447‡ | 0.344‡ |  |
| 0 morbidities | 154 | 0 | 3 | 16 | 56 | 100 | 60 (39%) | Ref | Ref | – |
| 1 morbidity | 299 | 0 | 2 | 14 | 48 | 113 | 106 (35%) | 0.86 (0.58–1.29) | 0.86 (0.57–1.30) | – |
| 2 morbidities | 288 | 0 | 2 | 13.5 | 40.5 | 76 | 94 (33%) | 0.76 (0.51–1.14) | 0.78 (0.51–1.20) | – |
| 3+ morbidities | 293 | 0 | 3 | 14 | 46 | 99 | 112 (38%) | 0.97 (0.65–1.45) | 1.05 (0.68–1.61) | – |
| Charlson score |  | 0.947† | | | | | | 0.627‡ | 0.590‡ | 0.490‡ |
| 0 | 6872 | 0 | 0 | 5 | 27 | 78 | 1646 (24%) | Ref | Ref | Ref |
| 1 | 1265 | 0 | 0 | 5 | 25 | 73 | 288 (23%) | 0.94 (0.81–1.08) | 0.92 (0.80–1.07) | 0.90 (0.78–1.05) |
| 2 | 669 | 0 | 0 | 4 | 29 | 71 | 170 (25%) | 1.08 (0.90–1.30) | 1.07 (0.89–1.29) | 1.06 (0.87–1.28) |
| 3+ | 596 | 0 | 0 | 6 | 27.5 | 67 | 142 (24%) | 0.99 (0.82–1.21) | 0.97 (0.80–1.19) | 0.96 (0.78–1.18) |
| Colorectal cancer | | 0.769† | | | | |  | 0.293‡ | 0.185‡ |  |
| 0 | 813 | 0 | 0 | 5 | 28 | 101 | 196 (24%) |  |  | – |
| 1 | 155 | 0 | 0 | 3 | 22 | 86 | 30 (19%) | 0.76 (0.49–1.16) | 0.73 (0.47–1.13) | – |
| 2 | 74 | 0 | 0 | 5.5 | 41 | 108 | 22 (30%) | 1.33 (0.79–2.25) | 1.42 (0.83–2.43) | – |
| 3+ | 75 | 0 | 0 | 4 | 23 | 141 | 15 (20%) | 0.79 (0.44–1.42) | 0.78 (0.42–1.42) | – |
| Lung cancer | | 0.462† | | | | |  | 0.668‡ | 0.758‡ |  |
| 0 | 647 | 0 | 2 | 14 | 49 | 103 | 239 (37%) |  |  | – |
| 1 | 175 | 0 | 3 | 14 | 43 | 95 | 64 (37%) | 0.98 (0.70–1.39) | 0.98 (0.69–1.40) | – |
| 2 | 100 | 0 | 3 | 13.5 | 39 | 77.5 | 31 (31%) | 0.77 (0.49–1.21) | 0.80 (0.50–1.26) | – |
| 3+ | 112 | 0 | 0 | 10 | 43.5 | 99 | 38 (34%) | 0.88 (0.57–1.34) | 0.88 (0.57–1.35) | – |

^Total n=9402, 80% of patients had complete information on the PCI

†Kruskal-Wallis test p-value at 50^th^ centile

‡Wald test for overall significance

*Model adjusted for number of morbidities, sex, age, ethnicity, and IMD

**Model adjusted for number of morbidities, sex, age, ethnicity, IMD, and cancer (to account for potential cancer case-mix differences within each group of diagnostic difficulty)

### S7.2 Morbidities and diagnostic interval, among patients diagnosed with lung and colorectal cancer only

|  |  | Centiles of interest | | | | | DI >60 days | Crude OR for DI >60 days | Adjusted OR* for DI >60 days | Adjusted OR** for DI >60 days |
| --- | --- | --- | --- | --- | --- | --- | --- | --- | --- | --- |
|  | N^ | 10^th^ | 25^th^ | 50^th^ | 75^th^ | 90^th^ | N (%) | OR (95% CI) | OR (95% CI) | OR (95% CI) |
| All patients | 10236 | 10 | 18 | 42 | 91 | 190 | 3831 (37%) | – | – | – |
| No of morbidities | | **<0.001†** | | | | |  | **<0.001‡** | **0.001‡** | **0.004‡** |
| 0 morbidities | 2476 | 9 | 15 | 38 | 82 | 177 | 861 (35%) | Ref | Ref | Ref |
| 1 morbidity | 3171 | 10 | 19 | 44 | 93 | 195 | 1216 (38%) | **1.17 (1.05–1.30)** | **1.15 (1.02–1.29)** | 1.11 (0.98–1.25) |
| 2 morbidities | 2485 | 10 | 18 | 42 | 85 | 182 | 902 (36%) | 1.07 (0.95–1.20) | 1.06 (0.93–1.20) | 1.04 (0.91–1.19) |
| 3+ morbidities | 2104 | 10 | 20 | 47 | 100 | 209 | 852 (40%) | **1.28 (1.13–1.44)** | **1.29 (1.13–1.48)** | **1.26 (1.10–1.45)** |
| Colorectal cancer | | 0.165† | | | | |  | **0.041‡** | **0.040‡** |  |
| 0 morbidities | 281 | 13 | 24 | 44 | 86 | 177 | 101 (36%) | Ref | Ref | – |
| 1 morbidity | 362 | 14 | 28 | 49.5 | 109 | 193 | 151 (42%) | 1.28 (0.93–1.76) | 1.32 (0.94–1.84) | – |
| 2 morbidities | 307 | 11 | 26 | 51 | 106 | 194 | 125 (41%) | 1.22 (0.88–1.71) | 1.31 (0.92–1.88) | – |
| 3+ morbidities | 269 | 13 | 27 | 60 | 114 | 210 | 129 (48%) | **1.64 (1.17–2.31)** | **1.75 (1.19–2.55)** | – |
| Lung cancer | | 0.251† | | | | |  | 0.451‡ | 0.313‡ |  |
| 0 morbidities | 186 | 14 | 26 | 45 | 82 | 137 | 73 (39%) | Ref | Ref | – |
| 1 morbidity | 368 | 14 | 26 | 52 | 93 | 203 | 157 (43%) | 1.15 (0.80–1.65) | 1.24 (0.85–1.80) | – |
| 2 morbidities | 366 | 13 | 24 | 44.5 | 84 | 170 | 146 (40%) | 1.03 (0.72–1.47) | 1.10 (0.75–1.61) | – |
| 3+ morbidities | 383 | 12 | 27 | 55 | 104 | 184 | 172 (45%) | 1.26 (0.88–1.80) | 1.37 (0.93–2.01) | – |
| Charlson score |  | **0.016†** | | | | |  | **0.044‡** | 0.092‡ | 0.055‡ |
| 0 | 7404 | 10 | 18 | 42 | 87 | 181 | 2721 (37%) | Ref | Ref | Ref |
| 1 | 1408 | 9 | 18 | 42.5 | 95.5 | 209 | 530 (38%) | 1.04 (0.92–1.17) | 1.03 (0.91–1.16) | 1.02 (0.90–1.15) |
| 2 | 747 | 10 | 20 | 47 | 107 | 212 | 305 (41%) | **1.19 (1.02–1.38)** | **1.17 (>1.00–1.37)** | **1.19 (1.01–1.40)** |
| 3+ | 677 | 10 | 19 | 46 | 106 | 213 | 275 (41%) | **1.18 (>1.00–1.38)** | 1.16 (0.99–1.37) | **1.19 (>1.00–1.41)** |
| Colorectal cancer | | 0.173† | | | | |  | 0.185‡ | 0.207‡ |  |
| 0 | 883 | 13 | 26 | 49 | 101 | 182 | 357 (40%) | Ref | Ref | – |
| 1 | 167 | 11 | 26 | 46 | 105 | 234 | 66 (40%) | 0.96 (0.69–1.35) | 0.95 (0.67–1.34) | – |
| 2 | 86 | 13 | 24 | 58.5 | 120 | 188 | 41 (48%) | 1.34 (0.86–2.09) | 1.36 (0.86–2.13) | – |
| 3+ | 83 | 13 | 29 | 62 | 142 | 210 | 42 (51%) | 1.51 (0.96–2.37) | 1.49 (0.94–2.37) | – |
| Lung cancer | | 0.770† | | | | |  | 0.753‡ | 0.682‡ |  |
| 0 | 799 | 15 | 27 | 50 | 88 | 161 | 341 (43%) | Ref | Ref | – |
| 1 | 239 | 9 | 22 | 48 | 91 | 206 | 93 (39%) | 0.86 (0.64–1.15) | 0.84 (0.62–1.13) | – |
| 2 | 126 | 11 | 26 | 49.5 | 110 | 205 | 54 (43%) | 1.01 (0.69–1.47) | 1.01 (0.69–1.49) | – |
| 3+ | 139 | 10 | 22 | 55 | 112 | 248 | 60 (43%) | 1.02 (0.71–1.47) | 1.02 (0.70–1.49) | – |

^Total n=10236, 87% of patients had complete information on the DI

†Kruskal-Wallis test p-value at 50th centile

‡Wald test for overall significance

*Model adjusted for number of morbidities, sex, age, ethnicity, and IMD

**Model adjusted for number of morbidities, sex, age, ethnicity, IMD, and cancer (to account for potential cancer case-mix differences within each group of diagnostic difficulty)

### S7.3 Morbidities and pre-referral consultations, among patients diagnosed with lung and colorectal cancer only

|  | N^ | 3 or more consultations  N (%) | Crude OR for 3 or more consultations OR (95% CI) | Adjusted OR* for 3 or more consultations  OR (95% CI) | Adjusted OR** for 3 or more consultations  OR (95% CI) |
| --- | --- | --- | --- | --- | --- |
| All patients | 11473 | 2837 (25%) | - | - | - |
| No. of NCDA morbidities | |  | **<0.001‡** | **<0.001‡** | **0.010‡** |
| 0 morbidities | 2839 | 599 (21%) | Ref | Ref | Ref |
| 1 morbidity | 3554 | 880 (25%) | **1.23 (1.09–1.38)** | 1.11 (0.98–1.25) | 1.03 (0.91–1.17) |
| 2 morbidities | 2760 | 675 (24%) | **1.21 (1.07–1.37)** | 1.04 (0.91–1.19) | 0.98 (0.85–1.13) |
| 3+ morbidities | 2320 | 683 (29%) | **1.56 (1.37–1.77)** | **1.31 (1.14–1.51)** | **1.21 (1.05–1.40)** |
| Colorectal cancer | |  | 0.754‡ | 0.793‡ |  |
| 0 morbidities | 312 | 82 (26%) | Ref | Ref | – |
| 1 morbidity | 402 | 103 (26%) | 0.97 (0.69–1.35) | 0.90 (0.63–1.28) | – |
| 2 morbidities | 334 | 81 (24%) | 0.90 (0.63–1.28) | 0.82 (0.56–1.21) | – |
| 3+ morbidities | 300 | 84 (28%) | 1.09 (0.76–1.56) | 0.91 (0.61–1.36) | – |
| Lung cancer | |  | 0.336‡ | 0.357‡ |  |
| 0 morbidities | 207 | 80 (39%) | Ref | Ref | – |
| 1 morbidity | 409 | 157 (38%) | 0.99 (0.70–1.39) | 1.02 (0.71–1.45) | – |
| 2 morbidities | 401 | 154 (38%) | 0.99 (0.70–1.40) | 1.03 (0.72–1.49) | – |
| 3+ morbidities | 407 | 178 (44%) | 1.23 (0.88–1.74) | 1.27 (0.88–1.84) | – |
| Charlson score |  |  | 0.05‡ | 0.896‡ | 0.982‡ |
| 0 | 8298 | 1997 (24%) | Ref | Ref | Ref |
| 1 | 1561 | 405 (26%) | 1.11 (0.98–1.25) | 1.03 (0.91–1.17) | 0.98 (0.86–1.12) |
| 2 | 844 | 224 (27%) | 1.14 (0.97–1.34) | 1.04 (0.89–1.23) | 0.98 (0.83–1.16) |
| 3+ | 770 | 211 (27%) | **1.19 (1.01–1.41)** | 1.05 (0.88–1.24) | 0.97 (0.82–1.16) |
| Colorectal cancer | |  | 0.917‡ | 0.693‡ |  |
| 0 | 969 | 255 (26%) | Ref | Ref | – |
| 1 | 184 | 44 (24%) | 0.88 (0.61–1.27) | 0.80 (0.55–1.17) | – |
| 2 | 98 | 25 (26%) | 0.96 (0.60–1.54) | 0.92 (0.57–1.50) | – |
| 3+ | 97 | 26 (27%) | 1.03 (0.64–1.64) | 0.89 (0.55–1.45) | – |
| Lung cancer | |  | 0.682‡ | 0.679‡ |  |
| 0 | 875 | 352 (40%) | Ref | Ref | – |
| 1 | 254 | 94 (37%) | 0.87 (0.65–1.16) | 0.87 (0.65–1.17) | – |
| 2 | 142 | 61 (43%) | 1.12 (0.78–1.60) | 1.13 (0.78–1.62) | – |
| 3+ | 153 | 62 (41%) | 1.01 (0.71–1.44) | 0.99 (0.69–1.42) | – |

^Total n=11473, 98% of patients had complete information on number of pre-referral consultations

‡Wald test for overall significance

*Model adjusted for number of morbidities, sex, age, ethnicity, and IMD

**Model adjusted for number of morbidities, sex, age, ethnicity, IMD, and cancer (to account for potential cancer case-mix differences within each group of diagnostic difficulty)

### S7.4 Morbidities and investigations, among patients diagnosed with lung and colorectal cancer only

|  | **N^** | **At least one investigation**  **N (%)** | **Crude OR for at least one investigation**  **OR (95% CI)** | **Adjusted OR* for at least one investigation**  **OR (95% CI)** | **Adjusted OR** for at least one Investigation**  **OR (95% CI)** |
| --- | --- | --- | --- | --- | --- |
| Total | 11230 | 6777 (60%) | - | - | - |
| No. of NCDA morbidities | |  | **<0.001‡** | 0.914‡ | 0.365‡ |
| 0 morbidities | 2762 | 1544 (56%) | Ref | Ref | Ref |
| 1 morbidity | 3487 | 2128 (61%) | **1.24 (1.12–1.37)** | 1.01 (0.90–1.12) | 0.91 (0.80–1.04) |
| 2 morbidities | 2703 | 1678 (62%) | **1.29 (1.16–1.44)** | 0.97 (0.86–1.10) | 0.89 (0.77–1.03) |
| 3+ morbidities | 2278 | 1427 (63%) | **1.32 (1.18–1.48)** | 0.97 (0.85–1.11) | 0.88 (0.76–1.03) |
| Colorectal cancer | |  | 0.950‡ | 0.887‡ |  |
| 0 morbidities | 306 | 202 (66%) | Ref | Ref | – |
| 1 morbidity | 389 | 251 (65%) | 0.94 (0.68–1.28) | 0.91 (0.65–1.26) | – |
| 2 morbidities | 331 | 216 (65%) | 0.97 (0.70–1.34) | 0.93 (0.66–1.32) | – |
| 3+ morbidities | 296 | 189 (64%) | 0.91 (0.65–1.27) | 0.86 (0.60–1.25) | – |
| Lung cancer | |  | 0.287‡ | 0.369‡ |  |
| 0 morbidities | 206 | 174 (84%) | Ref | Ref | – |
| 1 morbidity | 408 | 337 (83%) | 0.87 (0.55–1.38) | 0.81 (0.50–1.29) | – |
| 2 morbidities | 398 | 319 (80%) | 0.74 (0.47–1.17) | 0.71 (0.44–1.15) | – |
| 3+ morbidities | 410 | 323 (79%) | 0.68 (0.44–1.07) | 0.66 (0.41–1.08) | – |
| Charlson score |  |  | 0.630‡ | **0.004‡** | **<0.001‡** |
| 0 | 8102 | 4886 (60%) | Ref | Ref | Ref |
| 1 | 1541 | 917 (60%) | 0.97 (0.87–1.08) | **0.84 (0.75–0.95)** | **0.74 (0.65–0.85)** |
| 2 | 832 | 518 (62%) | 1.09 (0.94–1.26) | 0.93 (0.80–1.08) | 0.87 (0.73–1.04) |
| 3+ | 755 | 456 (60%) | >1.00 (0.86–1.17) | **0.80 (0.68–0.94)** | **0.72 (0.60–0.86)** |
| Colorectal cancer | |  | 0.158‡ | 0.135‡ |  |
| 0 | 948 | 624 (66%) | Ref | Ref | – |
| 1 | 177 | 105 (59%) | 0.76 (0.55–1.05) | 0.74 (0.53–1.03) | – |
| 2 | 102 | 72 (71%) | 1.25 (0.80–1.95) | 1.21 (0.77–1.91) | – |
| 3+ | 95 | 57 (60%) | 0.78 (0.51–1.20) | 0.76 (0.48–1.18) | – |
| Lung cancer | |  | **0.041‡** | 0.064‡ |  |
| 0 | 870 | 724 (83%) | Ref | Ref | – |
| 1 | 254 | 202 (80%) | 0.78 (0.55–1.11) | 0.78 (0.54–1.11) | – |
| 2 | 142 | 111 (78%) | 0.72 (0.47–1.12) | 0.74 (0.47–1.16) | – |
| 3+ | 156 | 116 (74%) | **0.58 (0.39–0.87)** | **0.59 (0.39–0.90)** | – |

^Total n=11230, 96% of patients had complete information on investigations

*Model adjusted for number of morbidities, sex, age, ethnicity, and IMD

**Model adjusted for number of morbidities, sex, age, ethnicity, IMD, and cancer (to account for potential cancer case-mix differences within each group of diagnostic difficulty)

### S7.5 Morbidities and emergency referral, among patients diagnosed with lung and colorectal cancer only

|  |  | Referral type | | |  |  |  |
| --- | --- | --- | --- | --- | --- | --- | --- |
|  |  | 2WW | Non-2WW | Emergency | Crude OR for Emergency referral | Adj OR* for Emergency referral | Adj OR** for Emergency referral |
|  | Total N^ | N (%) | N (%) | N (%) | OR (95% CI) | OR (95% CI) | OR (95% CI) |
| Total | 10703 | 7785 (73%) | 1928 (18%) | 990 (9%) | - | - | - |
| No of NCDA morbidities | |  |  |  | **<0.001‡** | **<0.001‡** | **<0.001‡** |
| 0 morbidities | 2686 | 2001 (74%) | 518 (19%) | 167 (6%) | Ref | Ref | Ref |
| 1 morbidity | 3346 | 2436 (73%) | 626 (19%) | 284 (8%) | **1.40 (1.15–1.71)** | **1.24 (1.01–1.53)** | 1.14 (0.92–1.42) |
| 2 morbidities | 2550 | 1873 (73%) | 419 (16%) | 258 (10%) | **1.70 (1.39–2.08)** | **1.36 (1.09–1.69)** | **1.28 (1.02–1.61)** |
| 3+ morbidities | 2121 | 1475 (70%) | 365 (17%) | 281 (13%) | **2.30 (1.88–2.82)** | **1.72 (1.37–2.15)** | **1.60 (1.26–2.02)** |
| Colorectal cancer | |  |  |  | 0.431‡ | 0.497‡ |  |
| 0 morbidities | 292 | 200 (68%) | 62 (21%) | 30 (10%) | Ref | Ref | - |
| 1 morbidity | 373 | 239 (64%) | 86 (23%) | 48 (13%) | 1.29 (0.79–2.09) | 1.40 (0.83–2.34) | - |
| 2 morbidities | 308 | 219 (71%) | 49 (16%) | 40 (13%) | 1.30 (0.79–2.16) | 1.39 (0.80–2.42) | - |
| 3+ morbidities | 268 | 170 (63%) | 58 (22%) | 40 (15%) | 1.53 (0.92–2.54) | 1.54 (0.87–2.73) | - |
| Lung cancer | |  |  |  | **0.026‡** | 0.193‡ |  |
| 0 morbidities | 180 | 130 (72%) | 23 (13%) | 27 (15%) | Ref | Ref | - |
| 1 morbidity | 352 | 264 (75%) | 38 (11%) | 50 (14%) | 0.94 (0.57–1.56) | 0.90 (0.54–1.51) | - |
| 2 morbidities | 341 | 229 (67%) | 39 (11%) | 73 (21%) | 1.54 (0.95–2.50) | 1.36 (0.82–2.26) | - |
| 3+ morbidities | 345 | 220 (64%) | 52 (15%) | 73 (21%) | 1.52 (0.94–2.47) | 1.28 (0.77–2.16) | - |
| Charlson score |  |  |  |  | **<0.001‡** | **<0.001‡** | **<0.001‡** |
| 0 | 7786 | 5796 (74%) | 1378 (18%) | 612 (8%) | Ref | Ref | Ref |
| 1 | 1469 | 1005 (68%) | 282 (19%) | 182 (12%) | **1.66 (1.39–1.98)** | **1.48 (1.24–1.77)** | **1.41 (1.16–1.70)** |
| 2 | 773 | 535 (69%) | 150 (19%) | 88 (11%) | **1.51 (1.19–1.91)** | **1.30 (1.02–1.66)** | 1.21 (0.94–1.56) |
| 3+ | 675 | 449 (67%) | 118 (17%) | 108 (16%) | **2.23 (1.79–2.79)** | **1.80 (1.43–2.26)** | **1.61 (1.26–2.06)** |
| Colorectal cancer | |  |  |  | 0.105‡ | 0.177‡ |  |
| 0 | 898 | 612 (68%) | 181 (20%) | 105 (12%) | Ref | Ref | - |
| 1 | 168 | 109 (65%) | 33 (20%) | 26 (15%) | 1.38 (0.87–2.20) | 1.41 (0.87–2.28) | - |
| 2 | 90 | 60 (67%) | 20 (22%) | 10 (11%) | 0.94 (0.47–1.88) | 0.92 (0.46–1.86) | - |
| 3+ | 85 | 47 (55%) | 21 (25%) | 17 (20%) | 1.89 (1.07–3.34) | 1.76 (0.97–3.21) | - |
| Lung cancer | |  |  |  | 0.028‡ | 0.063‡ |  |
| 0 | 748 | 544 (73%) | 83 (11%) | 121 (16%) | Ref | Ref | - |
| 1 | 218 | 136 (62%) | 33 (15%) | 49 (22%) | 1.50 (1.03–2.18) | 1.46 (1.00–2.13) | - |
| 2 | 121 | 86 (71%) | 15 (12%) | 20 (17%) | 1.03 (0.61–1.72) | 0.92 (0.54–1.56) | - |
| 3+ | 131 | 77 (59%) | 21 (16%) | 33 (25%) | **1.74 (1.12–2.71)** | **1.60 (1.01–2.53)** | - |

^Total n=10703. 2WW = two-week-wait urgent referrals for suspected cancer; non-2WW includes routine, urgent non-cancer, and private referrals; emergency includes emergency referrals; 1013 (9%) individuals whose referrals were categorised as patient self-referrals to A&E, “other”, “screen-detected”, and “not known” were excluded.

*Model adjusted for number of morbidities, sex, age, ethnicity, and IMD

**Model adjusted for number of morbidities, sex, age, ethnicity, IMD, and cancer (to account for potential cancer case-mix differences within each group of diagnostic difficulty)

## S8 The referral-to-diagnosis interval

The referral-to-diagnosis interval was estimated by subtracting the primary care interval from the diagnostic interval (Weller *et al*, 2012).


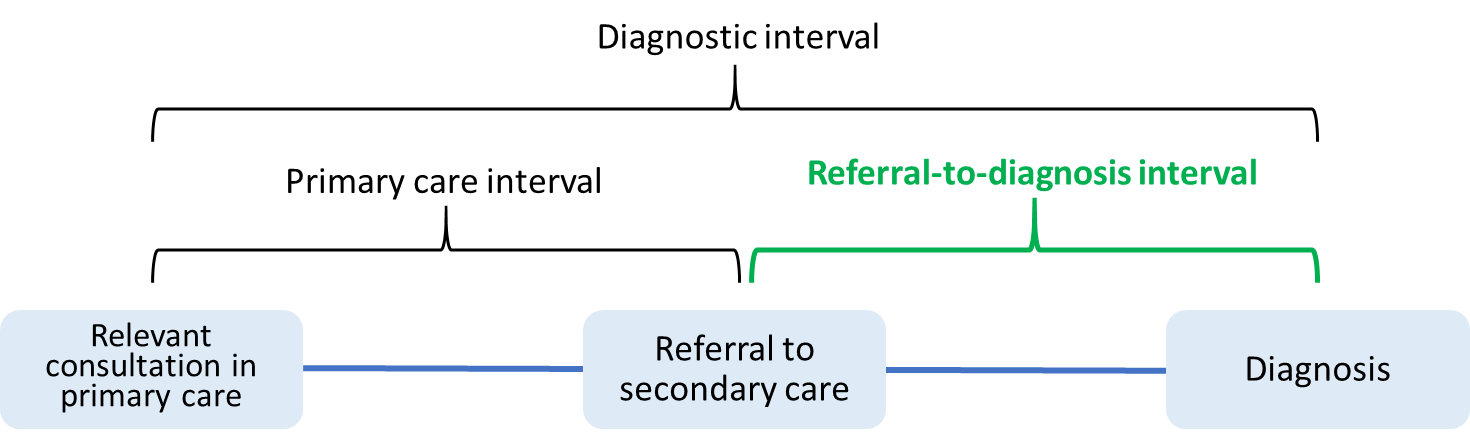


### S8.1 Descriptive statistics of the referral-to-diagnosis interval (RDI) by NCDA measured morbidity and Charlson score, and logistic regression output examining the outcome of a RDI >28 days

|  |  | Centiles of interest | | | | | RDI <=28 days | RDI >28 days | Crude OR for RDI >28 days | Adjusted OR* for RDI >28 days | Adjusted OR** for RDI >28 days |
| --- | --- | --- | --- | --- | --- | --- | --- | --- | --- | --- | --- |
|  | N^ | 10^th^ | 25^th^ | 50^th^ | 75^th^ | 90^th^ | N (%) | N (%) | OR (95% CI) | OR (95% CI) | OR (95% CI) |
| All patients | 9232 | 7 | 13 | 25 | 53 | 114 | 7156 (76%) | 2246 (24%) | – | – | – |
| No. of NCDA morbidities | | **0.001†** | | | | |  |  | **0.002‡** | **0.005‡** | **0.007‡** |
| 0 morbidities | 2297 | 7 | 12 | 22 | 49 | 102 | 1343 (58%) | 954 (42%) | Ref | Ref | Ref |
| 1 morbidity | 2876 | 7 | 13 | 25 | 53 | 117 | 1586 (55%) | 1290 (45%) | **1.15 (1.02–1.28)** | 1.12 (<1.00–1.26) | 1.10 (0.97–1.25) |
| 2 morbidities | 2234 | 7 | 13 | 25.5 | 52 | 112 | 1235 (55%) | 999 (45%) | **1.14 (1.01–1.28)** | 1.12 (0.99–1.27) | 1.12 (0.98–1.28) |
| 3+ morbidities | 1825 | 6 | 13 | 27 | 60 | 132 | 957 (52%) | 868 (48%) | **1.28 (1.13–1.45)** | **1.29 (1.12–1.48)** | **1.30 (1.12–1.50)** |
| Charlson score | | 0.947 | | | | |  |  | **0.010‡** | **0.034‡** | **0.013** |
| 0 | 6772 | 7 | 13 | 24 | 51 | 105 | 3823 (56%) | 2949 (44%) | Ref | Ref | Ref |
| 1 | 1239 | 6 | 13 | 26 | 57 | 140 | 667 (54%) | 572 (46%) | 1.11 (0.98–1.26) | 1.10 (0.97–1.24) | 1.12 (0.99–1.28) |
| 2 | 650 | 7 | 13 | 28 | 62 | 155 | 332 (51%) | 318 (49%) | **1.24 (1.06–1.46)** | **1.22 (1.04–1.44)** | **1.25 (1.05–1.49)** |
| 3+ | 571 | 6 | 12 | 27 | 60 | 145 | 299 (52%) | 272 (48%) | 1.18 (0.99–1.40) | 1.17 (0.98–1.39) | **1.21 (>1.00–1.46)** |

^Total n=9232, 79% of patients had complete information on the RDI

†Kruskal-Wallis test p-value at 50^th^ centile

‡Wald test for overall significance

*Model adjusted for number of morbidities, sex, age, ethnicity, and IMD

**Model adjusted for number of morbidities, sex, age, ethnicity, IMD, and cancer

[End of file]

1. Swann R, McPhail S, Witt J, et al. Diagnosing cancer in primary care: results from the National Cancer Diagnosis Audit. Br J Gen Pract. 2018;68(666):e63-e72. doi:10.3399/bjgp17X694169 [↑](#footnote-ref-1)
